# Triterpenoid Saponins from the Seeds of *Aesculus chinensis* and Their Cytotoxicities

**DOI:** 10.1007/s13659-017-0148-4

**Published:** 2017-12-29

**Authors:** Jin-Tang Cheng, Shi-Tao Chen, Cong Guo, Meng-Jiao Jiao, Wen-Jin Cui, Shu-Hui Wang, Zhe Deng, Chang Chen, Sha Chen, Jun Zhang, An Liu

**Affiliations:** Institute of Chinese Materia Medica, China Academy of Chinese Medical Sciences, Beijing, 100700 China

**Keywords:** *Aesculus chinensis*, Triterpenoid saponins, Chemical structures, Anti-tumor activity

## Abstract

**Abstract:**

Six new triterpenoid saponins, aesculusosides A–F (**1**–**6**), together with 19 known ones, were isolated from the seeds of *Aesculus chinensis*. The new structures were elucidated through extensive spectroscopic analyses and by comparison with previously reported data. Some of the isolates were evaluated for their cytotoxic activities against MCF-7 cell line by an MTT assay, and compounds **15**, **16**, **19**, and **23**–**25** exhibited inhibitory activities against MCF-7 with IC_50_ values ranging from 7.1 to 31.3 μM.

**Graphical Abstract:**

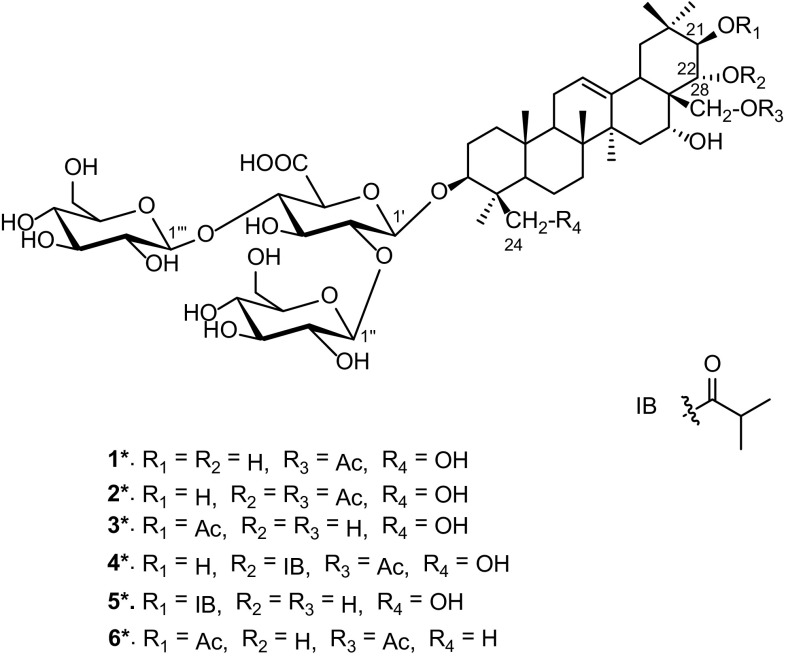

**Electronic supplementary material:**

The online version of this article (10.1007/s13659-017-0148-4) contains supplementary material, which is available to authorized users.

## Introduction

Hippocastanaceae is known to be a rich source of escins, a group of structurally diverse natural products characterized by a pentacyclic triterpenoid framework combined with a oligoglycoside chain [1]. Modern pharmacological studies show that some of these triterpenoid saponins possess diverse activities, including anti-inflammatory [2, 3], antitumor [4–7], antiviral [8], antioxidative [9, 10] and antigenotoxic properties [10].

**Fig. 1:**
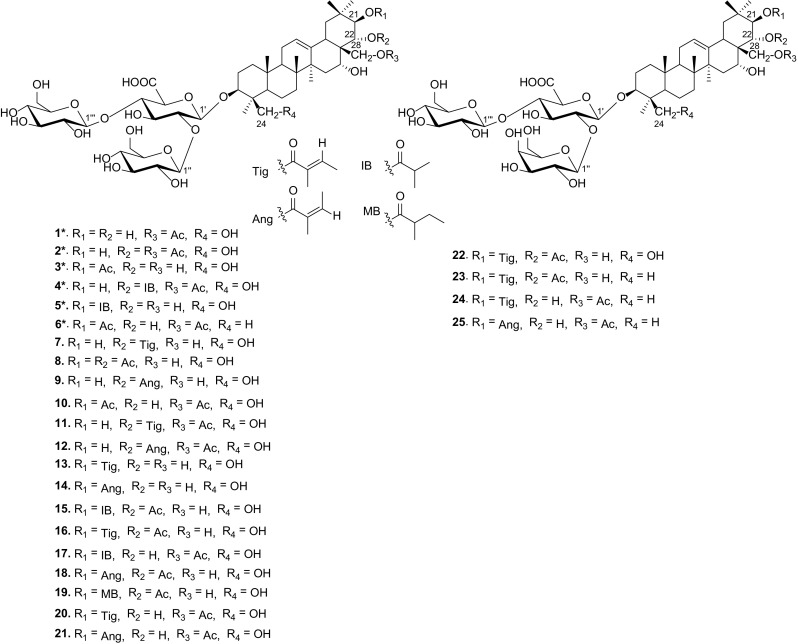
Structures of compounds **1**–**25**

*Aesculus chinensis* Bge. (Hippocastanaceae), abundant in the northwestern China, is a medicinal plant and its dried ripe seeds have been used as a stomachic and analgesic in the treatment of ditension and pain in the chest and abdomen [8]. Previous investigations on the chemical constituents of the seeds led to the isolation of an array of triterpenoid saponins [2, 8, 11–13]. In order to search for bioactive constituents from natural sources, we conducted the phytochemical investigation on the seeds of *A. chinensis* and identified six new (**1**–**6**) and 19 known triterpenoid saponins (**7**–**25**) (Fig. 1). Reported herein are the isolation and structure elucidation of compounds **1**–**6**, as well as the cytotoxicities of some isolates.

## Results and Discussion

**Fig. 2:**
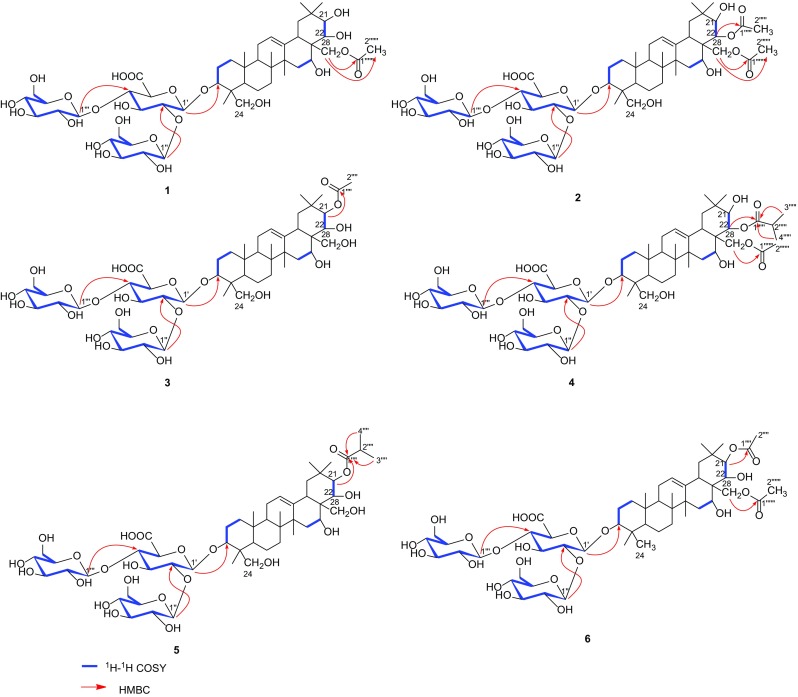
Key COSY and HMBC correlations of compounds **1**–**6**

Compound **1** was isolated as an amorphous powder, with the molecular formula of C_50_H_80_O_23_ as determined by its ^13^C-NMR data and negative-ion HR-ESI–MS (*m/z* 1047.5035 ([M−H]^−^), calc. 1047.5018). The IR absorption bands at 3424 and 1721 nm^−1^ implied the presence of the hydroxyls and carboxyl groups, respectively. In the ^1^H-NMR spectrum (Table 1), it displayed the occurrence of one olefinic proton (*δ*_H_ 5.28) and six methyls (*δ*_H_ 1.43, 1.21, 0.96, 0.93, 0.92, and 0.88), which was in conformity with the appearance of one olefinic C-atom (*δ*_C_ 129.9) and six methyls (*δ*_C_ 30.3, 27.6, 23.0, 19.2, 17.5, and 16.4) in its ^13^C-NMR data, characteristic of a triterpenoid skeleton [15]. In addition, the presence of three anomeric carbon signals at *δ*_C_ 104.9, 104.8 and 104.2, as well as other oxygenated carbon signals in the region of *δ*_C_ 83.0–61.9 in the ^13^C-NMR spectrum, indicated that there existed a trisaccharide moiety. The above evidence suggested that the structure of **1** showed a close resemblance to that of aesculuside-B [15], except for the presence of an additional acetyl group. Careful analysis of its HSQC and ^1^H-^1^H COSY spectra revealed the presence of the following fragments, a (C-1/C-2/C-3), b (C-15/C-16), c (C-21/C-22), d (C-1′/C-2′/C-3′/C-4′), e (C-1″/C-2″/C-3″/C-4″/C-5″/C-6″), and f (C-1′′′/C-2′′′/C-3′′′/C-4′′′/C-5′′′/C-6′′′) as shown in Fig. 2. As observed in the HMBC spectrum, a correlation between the signals of CH_2_ (*δ*_H_ 3.84 and 3.76, H_2_-28) and the acetyl carbons (*δ*_C_ 173.0 and 21.0) revealed that the acyl group was attached to C-28. Furthermore, the trisaccharidic structure was characterized by HMBC cross-peaks of H-1′ (*δ*_H_ 4.50)/C-3 (*δ*_C_ 92.5), H-1″ (*δ*_H_ 4.83)/C-2′ (*δ*_C_ 80.2) and H-1′′′ (*δ*_H_ 4.42)/C-4′ (*δ*_C_ 82.9). In the ROESY spectrum of **1**, there were no solid correlations which can be used to establish the relative configurations of C-3, C-16, C-21, and C-22. Thus, alkaline hydrolysis of **1** with 1% NaOMe, which liberated aesculuside-B, was conducted to assign the stereochemistry of chiral centers in **1** [15]. Furthermore, the coupling constant of H-21 and H-22 (*J* = 9.6 Hz) also supported the proposed stereochemistry of C-21 and C-22 [8]. Acid hydrolysis of **1** yielded glucose and glucuronic acid. The three monosaccharides were determined to be one *β*-d-glucuronopyranosyl acid and two *β*-d-glucopyranoses inferring from the coupling constants of the anomeric protons (*δ*_H_ 4.50, d, *J* = 7.6 Hz, H-1′; *δ*_H_ 4.83, d, *J* = 7.8 Hz, H-1″) and the typical carbon chemical shifts (*δ*_C_ 104.9, C-1′; *δ*_C_ 104.2, C-1″; *δ*_C_ 104.7, C-1′′′). Based on the above evidence, compound **1** was characterized as 28-*O*-acetylprotoaescigenin 3-*O*-[*β*-d-glucopyranosyl(1→2)][*β*-d-glucopyranosyl(1→4)]-*β*-d-glucopyranosiduronic acid.

**Table 1:** ^1^H-NMR data of compounds **1**–**6** recorded in methanol-*d*_*4*_ (*δ* in ppm, *J* in Hz, 600 MHz)

Position	**1**	**2**	**3**	**4**	**5**	**6**
1a	1.61, m	1.63, m	1.62, m	1.62, m	1.63, m	1.61, m
1b	0.98, m	1.00, m	0.99, m	0.99, m	1.00, m	0.99, m
2a	2.09, m	2.05, m	2.09, m	2.09, m	2.10, m	1.94, m
2b	1.81, d (12.7)	1.82, d (12.3)	1.82, d (12.3)	1.82, m	1.81, d (13.1)	1.71, d (12.7)
3	3.37, m	3.38, m	3.38, m	3.37, m	3.38, m	3.21, m
5	0.95, overlap	0.96, overlap	0.95, overlap	0.95, overlap	0.95, overlap	0.79, d (11.5)
6a	1.63, m	1.63, m	1.63, m	1.62, m	1.63, m	1.61, m
6b	1.36, m	1.36, m	1.37, m	1.37, m	1.36, m	1.43, m
7a	1.60, m	1.60, m	1.61, m	1.61, m	1.61, m	1.63, m
7b	1.36, overlap	1.36, overlap	1.36, overlap	1.36, m	1.36, overlap	1.37, d (12.9)
9	1.63, m	1.66, m	1.65, m	1.65, m	1.65, m	1.65, m
11	1.87, m, 2H	1.89, m, 2H	1.86, m, 2H	1.86, m, 2H	1.86, m, 2H	1.90, m, 2H
12	5.28, brs	5.33, brs	5.33, brs	5.34, t (3.4)	5.33, brs	5.31, brs
15a	1.76, m	1.71, dd (12.0, 3.2)	1.78, dd (14.7, 3.0)	1.69, m	1.77, dd (14.9, 3.2)	1.78, dd (14.7, 3.2)
15b	1.40, m	1.39, m	1.38, m	1.39, m	1.37, m	1.45, m
16	4.11, brs	4.11, brs	4.12, brs	4.10, brs	4.11, brs	4.11, brs
18	2.45, m	2.46, dd (13.9, 3.3)	2.42, dd (14.1, 3.3)	2.54, m	2.43, dd (14.1, 3.3)	2.49, dd (14.0, 3.4)
19a	2.47, m	2.57, t (13.5)	2.56, m	2.54, overlap	2.56, m	2.61, t (13.6)
19b	1.07, dd (12.0, 2.6)	1.12, m	1.12, d (4.5)	1.12, dd (12.4, 3.6)	1.11, dd (13.0, 4.0)	1.15, dd (12.9, 4.0)
21	3.94, d (9.6)	4.18, d (9.8)	5.50, d (10.0)	4.21, d (9.9)	5.49, d (10.0)	5.51, d (9.9)
22	3.71, d (9.6)	5.21, d (9.8)	3.96, d (10.0)	5.23, d (9.9)	3.96, d (10.0)	3.88, d (9.9)
23	1.20, s	1.21, s	1.21, s	1.20, s	1.20, s	1.10, s
24a	4.10, brs	4.10, brs	4.10, brs	4.10, brs	4.10, brs	0.87 (3H, s)
24b	3.21, m	3.21, m	3.21, m	3.21, m	3.20, m	
25	0.87, s	0.87, s	0.88, s	0.87, s	0.88, s	0.97, s
26	0.91, s	0.88, s	0.92, s	0.88, s	0.92, s	0.93, s
27	1.42, s	1.45, s	1.44, s	1.46, s	1.43, s	1.44, s
28a	3.84, t (11.2)	4.11, brs	3.34, m	3.64, m	3.32, m	3.87, m
28b	3.76, m	3.64, m	3.22, m	3.56, brs	3.22, m	3.74, m
29	0.96, s	0.97, s	0.83, s	0.96, s	0.82, s	0.85, s
30	0.92, s	0.98, s	0.98, s	0.99, s	0.99, s	1.00, s
1′	4.50, d (7.6)	4.52, d (7.2)	4.50, d (7.5)	4.49, d (7.8)	4.49, d (7.7)	4.50, d (6.6)
2′	3.62, m	3.62, m	3.62, m	3.62, m	3.61, m	3.70, m
3′	3.75, m	3.76, m	3.75, m	3.76, m	3.75, m	3.74, m
4′	3.63, m	3.71, m	3.63, m	3.64, m	3.62, m	3.71, m
5′	3.62, overlap	3.62, overlap	3.62, overlap	3.62, overlap	3.62, overlap	3.23, m
1″	4.83, d (7.8)	4.83, d (7.7)	4.83, d (7.7)	4.83, d (7.9)	4.83, d (7.7)	4.72, d (7.6)
2″	3.19, m	3.19, m	3.19, m	3.19, m	3.19, m	3.23, overlap
3″	3.35, m	3.35, m	3.35, m	3.35, m	3.35, m	3.35, m
4″	3.46, t (9.3)	3.46, t (9.3)	3.46, t (9.4)	3.46, t (9.4)	3.44, t (9.4)	3.32, m
5″	3.34, overlap	3.34, overlap	3.34, overlap	3.34, overlap	3.35, overlap	3.35, overlap
6a″	3.77, m	3.77, m	3.77, m	3.77, m	3.77, m	3.87, m
6b″	3.73, m	3.73, m	3.73, m	3.73, m	3.73, m	3.68, m
1′′′	4.42, brs	4.41, brs	4.42, brs	4.42, d (6.5)	4.42, d (6.2)	4.44, brs
2′′′	3.23, m	3.23, m	3.23, m	3.23, m	3.23, m	3.22, m
3′′′	3.38, m	3.38, m	3.38, m	3.38, m	3.38, m	3.36, m
4′′′	3.30, m	3.30, m	3.30, m	3.30, m	3.30, m	3.22, m
5′′′	3.31, m	3.34, m	3.36, m	3.35, m	3.35, m	3.23, m
6a′′′	3.87, d (11.2)	3.87, d (11.0)	3.87, d (11.6)	3.86, d (11.6)	3.85, d (11.9)	3.82, d (11.3)
6b′′′	3.66, m	3.66, m	3.66, m	3.66, m	3.64, m	3.62, m
21-*O*-moiety						
2″″			2.09, s		2.61, m	2.09, s
3″″					1.18, s	
4″″					1.20, s	
22-*O*-moiety						
2″″		2.08, s		2.62, m		
3″″				1.18, d (7.2)		
4″″				1.19, d (7.2)		
28-*O*-moiety						
2″″	2.05, s	2.02, s		2.04, s		2.05, s

Compound **2**, obtained as an amorphous powder, was assigned to have a molecular formula of C_52_H_82_O_24_ by negative-ion HR-ESI–MS (*m/z* 1089.5107 ([M-H]^−^), calc. 1089.5123). The ^1^H and ^13^C NMR data of **2** (Tables 1, 2) exhibited an identical trisaccharide moiety and aglycone as **1**. The only difference between them was an additional acetyl group in **2**, with an extra characteristic proton signal at *δ*_H_ 2.08 in its ^1^H-NMR spectrum and two additional carbon signals at *δ*_C_ 173.5, 21.4 in its ^13^C-NMR spectrum. Detailed analysis of its 1D and 2D NMR spectra suggested that the additional acetyl group was attached to C-22, which could be deduced by the key HMBC cross-peak of H-22 (*δ*_H_ 5.21)/C-1″″ (*δ*_C_ 173.5). The downfield shift of H-22 further supported the above conclusion. Therefore, **2** was established as 22,28-*O*-diacetyl-protoaescigenin 3-*O*-[*β*-d-glucopyranosyl-(1→2)][*β*-d-glucopyranosyl-(1→4)]-*β*-d-glucuronopyranosyl acid.

**Table 2:** ^13^C-NMR data of compounds **1**–**6** recorded in methanol-*d*_*4*_ (*δ* in ppm, 150 MHz)

Position	**1**	**2**	**3**	**4**	**5**	**6**
1	39.8, CH_2_	39.8, CH_2_	39.8, CH_2_	39.8, CH_2_	39.8, CH_2_	40.1, CH_2_
2	27.2, CH_2_	27.2, CH_2_	27.2, CH_2_	27.2, CH_2_	27.2, CH_2_	27.2, CH_2_
3	92.5, CH	92.6, CH	92.5, CH	92.5, CH	92.5, CH	91.6, CH
4	44.7, C	44.6, C	44.6, C	44.6, C	44.6, C	40.6, C
5	57.6, CH	57.5, CH	57.5, CH	57.5, CH	57.6, CH	57.1, CH
6	19.5, CH_2_	19.5, CH_2_	19.5, CH_2_	19.5, CH_2_	19.5, CH_2_	19.4, CH_2_
7	34.3, CH_2_	34.2, CH_2_	34.3, CH_2_	34.2, CH_2_	34.3, CH_2_	34.2, CH_2_
8	41.0, C	40.9, C	41.0, C	41.0, C	41.0, C	41.0, C
9	48.1, CH	47.9, CH	48.1, CH	48.0, CH	48.1, CH	48.1, CH
10	37.6, C	37.5, C	37.5, C	37.5, C	37.6, C	37.2, C
11	25.0, CH_2_	25.0, CH_2_	24.9, CH_2_	25.0, CH_2_	25.0, CH_2_	24.8, CH_2_
12	125.0, CH	125.4, CH	124.7, CH	125.4, CH	124.8, CH	125.4, CH
13	143.5, C	143.1, C	143.6, C	143.2, C	143.6, C	143.2, C
14	42.6, C	42.3, C	42.6, C	42.6, C	42.6, C	42.0, C
15	34.9, CH_2_	35.1, CH_2_	34.6, CH_2_	35.1, CH_2_	34.7, CH_2_	34.9, CH_2_
16	69.1, CH	69.1, CH	68.9, CH	69.4, CH	68.9, CH	68.9, CH
17	47.2, C	46.8, C	48.5, C	46.9, C	48.6, C	47.6, C
18	41.5, CH	42.1, CH	41.1, CH	42.1, CH	41.1, CH	41.3, CH
19	48.5, CH_2_	48.3, CH_2_	48.4, CH_2_	48.1, CH_2_	48.4, CH_2_	48.0, CH_2_
20	36.8, C	37.4, C	36.6, C	37.3, C	36.8, C	36.6, C
21	79.9, CH	77.6, CH	82.8, CH	77.7, CH	82.4, CH	82.6, CH
22	74.6, CH	78.2, CH	73.9, CH	76.9, CH	73.9, CH	72.3, CH
23	23.0, CH_3_	23.0, CH_3_	22.9, CH_3_	23.0, CH_3_	23.0, CH_3_	28.6, CH_3_
24	64.4, CH_2_	64.5, CH_2_	64.4, CH_2_	64.4, CH_2_	64.4, CH_2_	17.1, CH_2_
25	16.4, CH_3_	16.4, CH_3_	16.3, CH_3_	16.4, CH_3_	16.3, CH_3_	16.3, CH_3_
26	17.5, CH_3_	17.3, CH_3_	17.4, CH_3_	17.4, CH_3_	17.4, CH_3_	17.6, CH_3_
27	27.6, CH_3_	27.7, CH_3_	27.6, CH_3_	27.7, CH_3_	27.7, CH_3_	27.7, CH_3_
28	67.5, CH_2_	68.7, CH_2_	66.6, CH_2_	67.9, CH_2_	66.6, CH_2_	67.0, CH_2_
29	30.3, CH_3_	30.0, CH_3_	30.0, CH_3_	30.0, CH_3_	29.9, CH_3_	29.9, CH_3_
30	19.2, CH_3_	19.3, CH_3_	20.1, CH_3_	19.2, CH_3_	20.2, CH_3_	20.0, CH_3_
1′	104.9, CH	105.1, CH	104.9, CH	104.9, CH	104.9, CH	105.6, CH
2′	80.3, CH	80.0, CH	80.1, CH	80.1, CH	80.1, CH	80.4, CH
3′	77.6, CH	77.6, CH	77.5, CH	77.5, CH	77.5, CH	77.2, CH
4′	83.0, CH	82.3, CH	83.0, CH	82.9, CH	83.0, CH	82.9, CH
5′	77.6, CH	77.2, CH	77.5, CH	77.5, CH	77.5, CH	76.5, CH
6′	ND	ND	ND	ND	ND	172.3, C
1″	104.2, CH	104.2, CH	104.1, CH	104.1, CH	104.2, CH	104.7, CH
2″	75.7, CH	75.7, CH	75.6, CH	75.7, CH	75.7, CH	76.5, CH
3″	78.1^a^, CH	78.0^b^, CH	78.0^c^, CH	78.0^d^, CH	78.0^e^, CH	78.0^f^, CH
4″	70.3, CH	70.4, CH	70.4, CH	70.4, CH	70.4, CH	71.4, CH
5″	78.2, CH	78.3, CH	78.3, CH	78.3, CH	78.3, CH	78.3, CH
6″	61.9, CH_2_	62.0, CH_2_	61.9, CH_2_	61.9, CH_2_	61.9, CH_2_	62.6, CH_2_
1′′′	104.7, CH	104.6, CH	104.7, CH	104.8, CH	104.8, CH	104.6, CH
2′′′	75.3, CH	75.2, CH	75.2, CH	75.2, CH	75.2, CH	75.2, CH
3′′′	78.0^a^, CH	77.9^b^, CH	77.9^c^, CH	78.1^d^, CH	78.1^e^, CH	77.9^f^, CH
4′′′	71.4, CH	71.4, CH	71.3, CH	71.3, CH	71.3, CH	72.0, CH
5′′′	78.4, CH	78.4, CH	78.4, CH	78.4, CH	78.4, CH	78.4, CH
6′′′	62.6, CH_2_	62.6, CH_2_	62.6, CH_2_	62.6, CH_2_	62.6, CH_2_	63.2, CH_2_
21-*O*-moiety						
1″″			174.0, C		179.8, C	173.9, C
2″″			21.4, CH_3_		35.9, CH	21.4, CH_3_
3″″					19.5, CH_3_	
4″″					20.2, CH_3_	
22-*O*-moiety						
1″″		173.5, C		179.3, C		
2″″		21.4, CH_3_		35.7, CH		
3″″				19.5, CH_3_		
4″″				19.9, CH_3_		
28-*O*-moiety						
1″″	173.5, C	172.8, C		172.7, C		172.8, C
2″″	20.9, CH_3_	20.9, CH_3_		21.0, CH_3_		20.9, CH_3_

*ND* not detected

^a–f^Assignments may be interchanged

Compound **3** was determined to have the same molecular formula of C_50_H_80_O_23_ as **1** by its negative-ion HR-ESI–MS (*m/z* 1047.5005 ([M−H]^−^), calc. 1047.5018). The characteristic NMR data suggested that **3** had the similar structure as **1**. The significant differences in ^1^H and ^13^C-NMR spectra from those of **1** and **3** were chemical shifts of C-21(*δ*_C_ 82.8) and C-22 (*δ*_C_ 73.9) with corresponding protons at *δ*_H_ 5.50 and 3.97. The downfield shifts of C-21 implied that the acetyl group at the C-22 in **1** was transferred to C-21 in **3**, which was further confirmed by the key HMBC cross-peak of H-21(*δ*_H_ 5.50)/C-1″″ (*δ*_C_ 174.0). In addition, the alkaline hydrolysis of **3** yielded aesculuside-B [15]. Hence, the structure of **3** was determined as 21-*O*-acetylprotoaescigenin 3-*O*-[*β*-d-glucopyranosyl(1→2)][*β*-d-glucopyranosyl(1→4)]-*β*-d-glucopyranosiduronic acid.

The HR-ESI–MS data of **4** showed a [M−H]^−^ ion at *m/z* 1117.5425 (calc. 1117.5436), corresponding to a molecular formula of C_54_H_86_O_24_. The similar NMR data between **4** and **2** indicated that they are very close in the structure. Furthermore, the ^1^H-NMR spectrum of **4** displayed one methine proton (*δ*_H_ 2.62) and two doublet methyl protons (*δ*_H_ 1.18 and 1.19), with the corresponding carbon resonances at *δ*_C_ 35.7, 19.5 and 19.9 in its ^13^C-NMR spectrum. An isopropyl group was easily assembled by ^1^H-^1^H COSY correlations of H-2″″ (*δ*_H_ 2.62) with H-3″″ (*δ*_H_ 1.18) and H-4″″ (*δ*_H_ 1.19). The aforementioned data, together with the key HMBC cross-peaks of H-22 (*δ*_H_ 5.23)/C-1″″ (*δ*_C_ 179.3), H-2″″ (*δ*_H_ 2.62)/C-1″″ (*δ*_C_ 179.3), H-3″″ (*δ*_H_ 1.18)/C-1″″ (*δ*_C_ 179.3) and H-4″″ (*δ*_H_ 1.19)/C-1″″ (*δ*_C_ 179.3), suggested that the isopropyl group in **4** was installed at the C-1″″. Based on the above evidence, compound **4** was characterized as 22-*O*-isobutyryl-28-*O*-acetylprotoaescigenin 3-*O*-[*β*-d-glucopyranosyl(1→2)][*β*-d-glucopyranosyl(1→4)]-*β*-d-glucopyranosiduronic acid.

Compound **5** was isolated as an amorphous powder. Its molecular formula was determined by negative HR-ESI–MS [*m/z* 1075.5406 (calc. 1075.5331)] as C_52_H_84_O_23_, requiring 11 degrees of unsaturation. Careful comparison of the ^1^H and ^13^C-NMR spectra of **5** with those of **4** indicated a high degree of similarity. The differences between **5** and **4** were the absence of an acetic group at the C-28 and that the isopropyl group was transferred to C-21 other than C-22 in compound **4**, as confirmed by the combinational analysis of 2D NMR data (Fig. 2). Thus, compound **5** was elucidated as 21-*O*-isobutyrylprotoaescigenin 3-*O*-[*β*-d-glucopyranosyl(1→2)][*β*-d-glucopyranosyl(1→4)]-*β*-d-glucopyranosiduronic acid.

Compound **6** was obtained as an amorphous powder and gave a molecular formula of C_52_H_82_O_23_, as determined by HR-ESI–MS at *m/z* 1073.5162 (calc. 1073.5174). The ^1^H-NMR spectrum of **6** (Table 1) revealed nine methyls [*δ*_H_ 0.84 (3H, s), 0.86 (3H, s), 0.93 (3H, s), 0.97 (3H, s), 1.00 (3H, s), 1.09 (3H, s), 1.44 (3H, s), 2.06 (3H, s), 2.09 (3H, s)] and one olefinic proton (*δ*_H_ 5.31).The above data, together with its ^13^C-NMR data (Table 2), indicated that aglycone moiety in **6** was barringtogenol C [16]. Furthermore, the characteristic carbon resonances displayed in the region of *δ*_C_ 83.0–62.6 in its ^13^C-NMR data (Table 2), indicated that there also existed a trisaccharide moiety. Acid hydrolysis of **6** also yielded glucose and glucuronic acid. The HMBC correlations from H-21 to C-20/C-22/C-29/C-30/C-1″″, and from H-28 to C-1″″, allowed for the assignment of two acetyl groups as shown in Fig. 1. Hence, the structure of **6** was deduced as 21,28-*O*-diacetylbarringtogenol C 3-*O*-[*β*-d-glucopyranosyl (1→2)][*β*-d-glucopyranosyl(1→4)]-*β*-d-glucopyranosiduronic acid.

The 19 known compounds were identified as 22-*O*-tigloylprotoaescigenin 3-*O*-[*β*-d-glucopyranosyl(1→2)][*β*-d-glucopyranosyl(1→4)]-*β*-d-glucopyranosiduronic acid [Escin Ivg] (**7**) [17], 21,22-*O*-diacetylprotoaescigenin 3-*O*-[*β*-d-glucopyranosyl(1→2)][*β*-d-glucopyranosyl(1→4)]-*β*-d-glucopyranosiduronic acid [Escin Iv] (**8**) [18], 22-*O*-angeloylprotoaescigenin 3-*O*-[*β*-d-glucopyranosyl(1→2)][*β*-d-glucopyranosyl(1→4)]-*β*-d-glucopyranosiduronic acid [Escin Ivh] (**9**) [2], 21,28-*O*-diacetylprotoaescigenin 3-*O*-[*β*-d-glucopyranosyl (1→2)][*β*-d-glucopyranosyl(1→4)]-*β*-d-glucopyranosiduronic acid [Aesculiside A] (**10**) [19], 22-*O*-tigloyl-28-*O*-acetylprotoaescigenin 3-*O*-[*β*-d-glucopyranosyl(1→2)][*β*-d-glucopyranosyl(1→4)]-*β*-d-glucopyranosiduronic acid [Escin IVc] (**11**) [20], 22-*O*-angeloyl-28-*O*-acetylprotoaescigenin 3-*O*-[*β*-d-glucopyranosyl (1→2)] [*β*-d-glucopyranosyl(1→4)]-*β*-d-glucopyranosiduronic acid [Escin IVd] (**12**) [20], 21-*O*-tigloylprotoaescigenin 3-*O*-[*β*-d-glucopyranosyl(1→2)][*β*-d-glucopyranosyl(1→4)]-*β*-d-glucopyranosiduronic acid [Aesculioside A] (**13**) [13], 21-*O*-angeloylprotoaescigenin 3-*O*-[*β*-d-glucopyranosyl(1→2)][*β*-d-glucopyranosyl(1→4)]-*β*-d-glucopyranosiduronic acid [Aesculioside B] (**14**) [13], 21-*O*-isobutyryl-22-*O*-acetylprotoaescigenin 3-*O*-[*β*-d-glucopyranosyl(1→2)][*β*-d-glucopyranosyl(1→4)]-*β*-d-glucopyranosiduronic acid [Escin V] (**15**) [18], 21-*O*-tigloyl-22-*O*-acetylprotoaescigenin 3-*O*-[*β*-d-glucopyranosyl (1→2)][*β*-d-glucopyranosyl(1→4)]-*β*-d-glucopyranosiduronic acid (Escin Ia) (**16**) [20], 21-*O*-isobutyryl-28-*O*-acetylprotoaescigenin 3-*O*-[*β*-d-glucopyranosyl(1→2)][*β*-d-glucopyranosyl(1→4)]-*β*-d-glucopyranosiduronic acid [Isoescin V] (**17**) [18], 21-*O*-angeloyl-22-*O*-acetylprotoaescigenin 3-*O*-[*β*-d-glucopyranosyl (1→2)] [*β*-d-glucopyranosyl(1→4)]-*β*-d-glucopyranosiduronic acid (Escin Ib) (**18**) [21], 21-*O*-isovaleryl-22-*O*-acetylprotoaescigenin 3-*O*-[*β*-d-glucopyranosyl (1→2)][*β*-d-glucopyranosyl(1→4)]-*β*-d-glucopyranosiduronic acid [Esvin VI] (**19**) [18], 21-*O*-tigloyl-28-*O*-acetylprotoaescigenin 3-*O*-[*β*-d-glucopyranosyl (1→2)] [*β*-d-glucopyranosyl(1→4)]-*β*-d-glucopyranosiduronic acid (isoescin Ia) (**20**) [20], 21-*O*-angeloyl-28-*O*-acetylprotoaescigenin 3-*O*-[*β*-d-glucopyranosyl (1→2)][*β*-d-glucopyranosyl(1→4)]-*β*-d-glucopyranosiduronic acid (isoescin Ib) (**21**) [20], 21-*O*-tigloyl-22-*O*-acetylprotoaescigenin 3-*O*-[*β*-d-galactopyranosyl(1→2)] [*β*-d-glucopyranosyl(1→4)]-*β*-d-glucopyranosiduronic acid (**22**) [22], 21-*O*-tigloyl-22-*O*-acetylbarringtogenol C 3-*O*-[*β*-d-glucopyranosyl (1→2)][*β*-d-glucopyranosyl(1→4)]-*β*-d-glucopyranosiduronic acid [Escin IIIa] (**23**) [21], 21-*O*-tigloyl-28-*O*-acetylbarringtogenol C 3-*O*-[*β*-d-glucopyranosyl (1→2)][*β*-d-glucopyranosyl(1→4)]-*β*-d-glucopyranosiduronic acid [Isoescin IIIa] (**24**) [21], 21-*O*-angeloyl-28-*O*-acetylbarringtogenol C 3-*O*-[*β*-d-glucopyranosyl (1→2)][*β*-d-glucopyranosyl(1→4)]-*β*-d-glucopyranosiduronic acid [Isoescin IIIb] (**25**) [11].

The known compounds **7**-**9**, **12**-**17**, **19**, and **22**–**25** were evaluated for their in vitro cytotoxicities against human cancer cell line (MCF-7) using the MTT method as reported previously [14], with *cis*-platin as positive control (Table 3). Compound **23** showed significant toxicity effect against MCF-7 with IC_50_ value of 7.1 μM, while **15** and **24** showed moderate cytotoxicities against MCF-7 cell line, with IC_50_ values of 16.9 and 11.8 μM, respectively.

**Table 3:** Cytotoxicities of compounds **7**–**9**, **12**–**17**, **19**, and **22**–**25** against MCF-7 tumor cell line with IC_50_ (μM)

Compound	IC_50_ (μM)
**7**	> 40
**8**	> 40
**9**	> 40
**12**	> 40
**13**	> 40
**14**	> 40
**15**	16.9
**16**	31.3
**17**	> 40
**19**	23.9
**22**	> 40
**23**	7.1
**24**	11.8
**25**	29.5
*Cis*-platin^a^	13.0

^a^Positive control

## Experimental Section

### General Experimental Procedures

Optical rotations were measured with Perkin Elmer/Model-343 digital polarimeter. IR spectra were recorded on a JASCO FT/IR-480 spectrophotometer and reported as wave number (cm^−1^). ^1^H, ^13^C NMR spectra and 2D NMR spectra were recorded on a Bruker Avance III 600 spectrometer. Chemical shifts were reported using TMS as the internal standard. HR-ESI–MS data were obtained on a Bruker Apex IV FT-MS spectrometer. Column chromatography (CC) was carried out using D-101 macroreticular resin (Tianjin Polymer Technology Co. Ltd.), MCI gel (75–150 μm, Mitsubishi Chemical Industries, Japan) and silica gel (90–200 µm; Qingdao Marine Chemical Co. Ltd., Qingdao, People’s Republic of China). MPLC was performed on a Lisui EZ Purify III System packed with RP-18 silica gel (40–63 μm, Merck, 71 Darmstadt, Germany) columns. Precoated silica gel GF254 plates (Qingdao Marine Chemical Co. Ltd, Qingdao, People’s Republic of China) were used for thin-layer chromatography (TLC). Preparative HPLC was performed on Shimadzu LC-8A equipped with a Shimadzu PRC-ODS(K) column and Agilent 1100 apparatus equipped with a Zorbax SB-C-1875 (Agilent, 9.4 mm × 25 cm) column, respectively.

### Plant Material

The seeds of *A. chinensis* were collected from Enshi, Hubei Province, P. R. China in October 2014, and were identified by Dr. Wei Sun (Institute of Chinese Materia Medica, China Academy of Chinese Medical Sciences). A voucher specimen (201410 M) was deposited in the herbarium at the department of medicinal plants, Institute of Chinese Materia Medica, China Academy of Chinese Medical Sciences (Beijing 100700, China).

### Extraction and Isolation

The dried seeds of *A. chinensis* (10 kg) were extracted with 70% ethanol under reflux for three times (3, 2, and 1 h, respectively). The resultant extract was resolved in H_2_O and extracted with EtOAc for three times. After removal of the EtOAc fraction, the remaining solution was then extracted with butanol for three times. The butanol solution was concentrated under reduced pressure and the butanol fraction (56 g) was subjected to D-101 (eluted with 20% ethanol, 40% ethanol, 70% ethanol and 95% ethanol) to afford four fractions. The 40% ethanol-eluted fraction (12 g) was decolorized over MCI gel (eluted with 90% MeOH) and then was subjected to MPLC (MeOH/H_2_O 5–35%) to provide Frs. 1–5. Frs. 1 (4.8 g) was further separated by using repeated prep. HPLC (MeOH/H_2_O 8–20%) to give five subfractions, Frs. 1A-1E. Subfraction Frs. 1A (0.4 g) was purified by semipreparative HPLC (MeCN/H_2_O, 11%) to afford compounds **1** (11 mg), **7** (8 mg), **8** (23 mg), and **9** (4 mg). Compounds **2** (5 mg), **3** (4 mg), **10** (16 mg), **11** (5 mg), **12** (7 mg), and **13** (8 mg) were obtained by two times of semipreparative HPLC (MeCN/H_2_O, 13%) from Frs. 1B (0.6 g). Subfraction Frs. 1C (0.1 g) was separated by semipreparative HPLC (MeOH/H_2_O, 15%) to afford **4** (2 mg) and **5** (1 mg). Subfraction Frs. 1D (0.3 g) was subjected to semipreparative HPLC (MeOH/H_2_O, 18%) to give compounds **6** (8 mg), **14** (5 mg), **15** (2 mg), and **22** (4 mg). Compounds **16** (6 mg), **17** (11 mg), and **18** (15 mg) were obtained from Frs. 2 by repeated prep. HPLC purification. Frs. 3 was subjected to repeated prep. HPLC and semi-perp. HPLC to afford compounds **19** (5 mg) and **23** (2 mg). From Frs. 4 and Frs. 5, compounds **20** (4 mg), **21** (4 mg), **24** (3 mg) and **25** (1 mg) were obtained by repeated prep. HPLC and semi-perp. HPLC.

#### Aesculusoside A (**1**)

Amorphous powder; [*α*]_D_^25^ − 2.5 (*c* 0.1, MeOH); IR (KBr) 3423, 2951, 1721, 1380, 1261, 1031 cm^−1^; ^1^H and ^13^C NMR data: see Tables 1 and 2; HR-ESI–MS (*m/z* 1047.5035 ([M−H]^−^), calc. 1047.5018).

#### Aesculusoside B (**2**)

Amorphous powder; [*α*]_D_^25^ − 3.5 (*c* 0.1, MeOH); IR (KBr) 3421, 2945, 1725, 1384, 1272, 1077 cm^−1^; ^1^H and ^13^C NMR data: see Tables 1 and 2; HR-ESI–MS (*m/z* 1089.5107 ([M−H]^−^), calc. 1089.5123).

#### Aesculusoside C (**3**)

Amorphous powder; [*α*]_D_^25^ − 34.5 (*c* 0.1, MeOH); IR (KBr) 3422, 2928, 1720, 1383, 1273, 1076 cm^−1^; ^1^H and ^13^C NMR data: see Tables 1 and 2; HR-ESI–MS (*m/z* 1047.5005 ([M−H]^−^), calc. 1047.5018).

#### Aesculusoside D (**4**)

Amorphous powder; [*α*]_D_^25^ − 32.1 (*c* 0.1, MeOH); IR (KBr) 3421, 2946, 1723, 1383, 1277, 1074 cm^−1^; ^1^H and ^13^C NMR data: see Tables 1 and 2; HR-ESI–MS (*m/z* 1117.5425 ([M−H]^−^), (calc. 1117.5436).

#### Aesculusoside E (**5**)

Amorphous powder; [*α*]_D_^25^ − 2.5 (*c* 0.1, MeOH);) IR (KBr) 3423, 2930, 1721, 1375, 1277, 1075 cm^−1^; ^1^H and ^13^C NMR data: see Tables 1 and 2; HR-ESI–MS (*m/z* 1075.5406 ([M−H]^−^), (calc. 1075.5331).

#### Aesculusoside F (**6**)

Amorphous powder; [*α*]_D_^25^ − 2.8 (*c* 0.1, MeOH); IR (KBr) 3423, 2929, 1721, 1384, 1273, 1075 cm^−1^; ^1^H and ^13^C NMR data: see Tables 1 and 2; HR-ESI–MS (1073.5162 ([M−H]^−^), (calc. 1073.5174).

### Cytotoxicity Assay

Compounds were tested in vitro for their cytotoxicities against proliferation of MCF-7 (breast cancer) using the MTT method [14]. The human tumor cell line MCF-7 was obtained from ATCC (Manassas, VA, USA). All cells were cultured in DMEM medium (Biological Industries, Kibbutz Beit-Haemek, Israel), which were supplemented with 10% fetal bovine serum (Biological Industries, Kibbutz Beit-Haemek, Israel) at 37 °C in a humidified atmosphere containing 5% CO_2_. Briefly, cells were seeded into each well of a 96-well cell culture plate. After 12 h of incubation at 37 °C, the test compound (40 μM) was added. After incubation for 48 h, cells were subjected to the MTT assay. Compounds with a growth inhibition rate of 50% were further evaluated with *cis*-platin (Sigma, St. Louis, MO, USA) as positive control.

### Acid Hydrolysis of Compounds **1** and **6**

A solution of compound **1** (4 mg) in H_2_O (1 mL) was treated with 20% aqueous H_2_SO_4_ (1 mL), and the mixture was heated under reflux for 2 h. It was then neutralized by saturated NaHCO_3_ and extracted three times with EtOAc. Glucose and glucuronic acid were obtained from the H_2_O layer, and identified by comparison with authentic samples and by PC (Paper Chromatography) behavior, solvent: CHCl_3_-MeOH-H_2_O, 10:6:1.

### Alkaline Hydrolysis of Compound **1**

Compound **1** (5 mg) was added to a MeOH solution (5 ml) of NaOMe (1 mg). The mixture was stirred at room temperature for 4 h and then neutralized with 20% aqueous HCl. The reaction mixture was concentrated under reduced pressure, and the residue was purified by column chromatography on silica gel (CH_2_Cl_2_/MeOH 2:1–1:1) to furnish aesculuside-B (1.2 mg, 46% yield), which was identical with authentic sample by TLC, ^1^H- and ^13^C-NMR spectra comparisons [15].

## Electronic supplementary material

Below is the link to the electronic supplementary material.

Supplementary material 1 (DOCX 2192 kb)
