# Supplementary material for: Triterpenoid Saponins from the Seeds of Aesculus chinensis and Their Cytotoxicities
Source: Nat Prod Bioprospect. 2017 Dec 29;8(1):47–56. doi: 10.1007/s13659-017-0148-4 (PMC5803144; doi:10.1007/s13659-017-0148-4)
Supplement: Supplementary file 1 — Supplementary material 1 (DOCX 2192 kb) [file 13659_2017_148_MOESM1_ESM.docx]

**Supporting Information**

**Triterpenoid Saponins from the Seeds of *Aesculus chinensis* and Their Cytotoxicities**

Jin-Tang Cheng • Shi-Tao Chen • Cong Guo • Meng-Jiao Jiao • Wen-Jin Cui • Shu-Hui Wang • Zhe Deng • Chang Chen • Sha Chen • Jun Zhang • An Liu (🖂)

Institute of Chinese Materia Medica, China Academy of Chinese Medical Sciences, Beijing 100700, China

Jin-Tang Cheng and Shi-Tao Chen contributed equally to this work.

E-mail address: la62@163.com (A. Liu)

**Contents of Supporting Information**

[Figure S1. ^1^H-NMR spectrum of compound **1** S5](#_Toc493160754)

[Figure S2. DEPT 90 and ^13^C-NMR spectrum of compound **1** S6](#_Toc493160755)

[Figure S3. DEPT 135 spectrum of compound **1** S6](#_Toc493160756)

[Figure S4. HSQC spectrum of compound **1** S7](#_Toc493160757)

[Figure S5. ^1^H-^1^H COSY spectrum of compound **1** S8](#_Toc493160758)

[Figure S6. HMBC spectrum of compound **1** S9](#_Toc493160759)

[Figure S7. NOESY spectrum of compound **1** S10](#_Toc493160760)

[Figure S8. HR-ESI-MS of compound **1** S11](#_Toc493160761)

[Figure S9. ^1^H-NMR spectrum of compound **2** S12](#_Toc493160762)

[Figure S10. DEPT 90 and ^13^C-NMR spectrum of compound **2** S13](#_Toc493160763)

[Figure S11. DEPT 135 spectrum of compound **2** S13](#_Toc493160764)

[Figure S12. HSQC spectrum of compound **2** S14](#_Toc493160765)

[Figure S13. ^1^H-^1^H COSY spectrum of compound **2** S15](#_Toc493160766)

[Figure S14. HMBC spectrum of compound **2** S16](#_Toc493160767)

[Figure S15. NOESY spectrum of compound **2** S17](#_Toc493160768)

[Figure S16. HR-ESI-MS of compound **2** S18](#_Toc493160769)

[Figure S17. ^1^H-NMR spectrum of compound **3** S19](#_Toc493160770)

[Figure S18. DEPT 90 and ^13^C-NMR spectrum of compound **3** S20](#_Toc493160771)

[Figure S19. DEPT 135 spectrum of compound **3** S20](#_Toc493160772)

[Figure S20. HSQC spectrum of compound **3** S21](#_Toc493160773)

[Figure S21. ^1^H-^1^H COSY spectrum of compound **3** S22](#_Toc493160774)

[Figure S22. HMBC spectrum of compound **3** S23](#_Toc493160775)

[Figure S23. NOESY spectrum of compound **3** S24](#_Toc493160776)

[Figure S24. HR-ESI-MS of compound **3** S25](#_Toc493160777)

[Figure S25. ^1^H-NMR spectrum of compound **4** S26](#_Toc493160778)

[Figure S26. DEPT 90 and ^13^C-NMR spectrum of compound **4** S27](#_Toc493160779)

[Figure S27. DEPT 135 spectrum of compound **4** S27](#_Toc493160780)

[Figure S28. HSQC spectrum of compound **4** S28](#_Toc493160781)

[Figure S29. ^1^H-^1^H COSY spectrum of compound **4** S29](#_Toc493160782)

[Figure S30. HMBC spectrum of compound **4** S30](#_Toc493160783)

[Figure S31. HR-ESI-MS of compound **4** S31](#_Toc493160784)

[Figure S32. ^1^H-NMR spectrum of compound **5** S32](#_Toc493160785)

[Figure S33. DEPT 90 and ^13^C-NMR spectrum of compound **5** S33](#_Toc493160786)

[Figure S34. DEPT 135 spectrum of compound **5** S33](#_Toc493160787)

[Figure S35. HSQC spectrum of compound **5** S34](#_Toc493160788)

[Figure S36. ^1^H-^1^H COSY spectrum of compound **5** S35](#_Toc493160789)

[Figure S37. HMBC spectrum of compound **5** S36](#_Toc493160790)

[Figure S38. NOESY spectrum of compound **5** S37](#_Toc493160791)

[Figure S39. HR-ESI-MS of compound **5** S38](#_Toc493160792)

[Figure S40. ^1^H-NMR spectrum of compound **6** S39](#_Toc493160793)

[Figure S41. DEPT 90 and ^13^C-NMR spectrum of compound **6** S40](#_Toc493160794)

[Figure S42. DEPT 135 spectrum of compound **6** S40](#_Toc493160795)

[Figure S43. HSQC spectrum of compound **6** S41](#_Toc493160796)

[Figure S44. ^1^H-^1^H COSY spectrum of compound **6** S42](#_Toc493160797)

[Figure S45. HMBC spectrum of compound **6** S43](#_Toc493160798)

[Figure S46. NOESY spectrum of compound **6** S44](#_Toc493160799)

[Figure S47. HR-ESI-MS of compound **6** S45](#_Toc493160800)


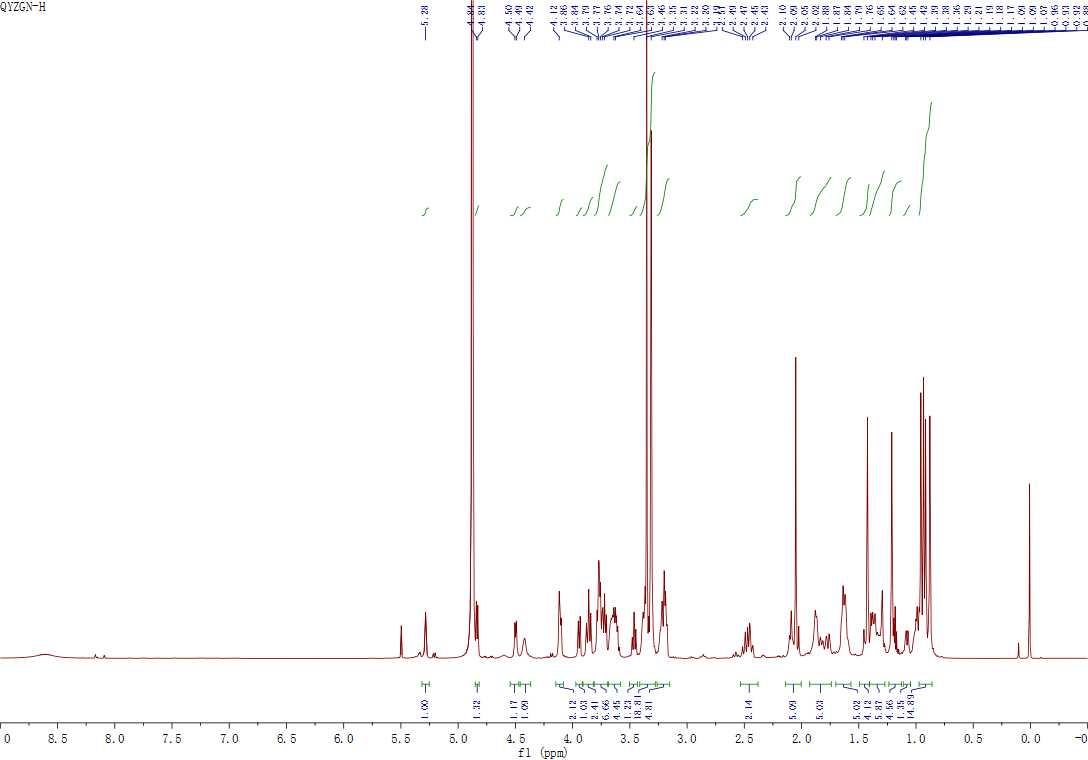


# Figure S1. ^1^H-NMR spectrum of compound 1


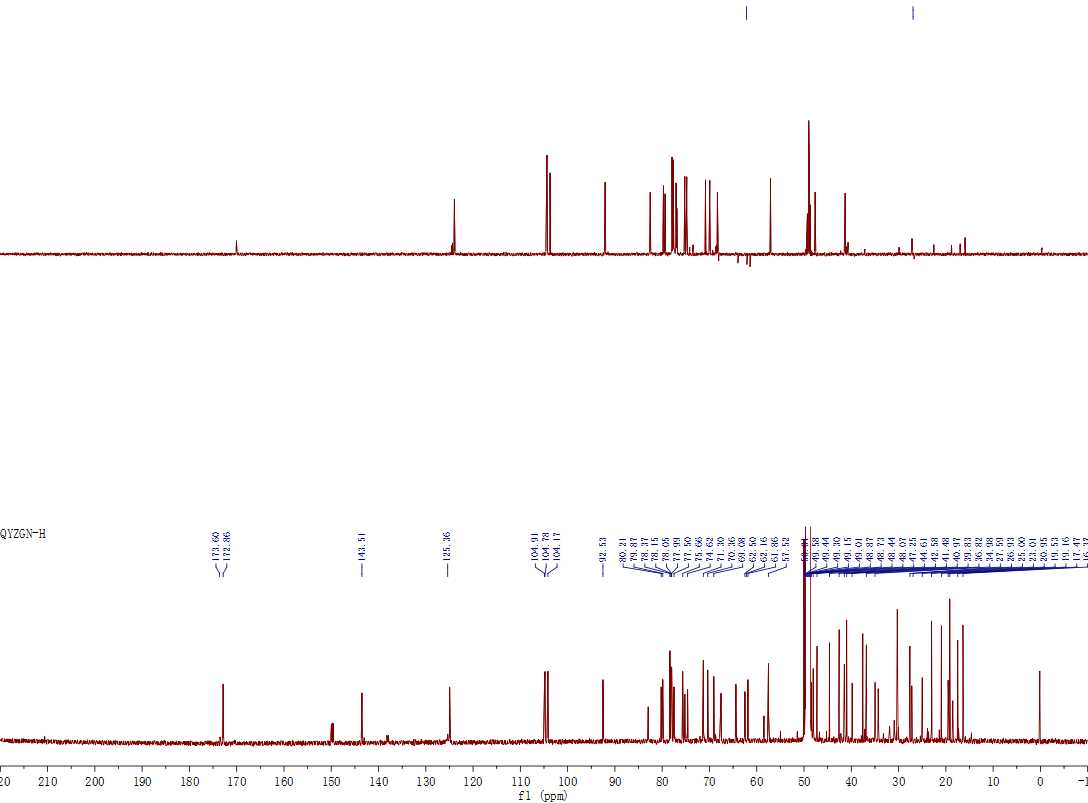


# Figure S2. DEPT 90 and ^13^C-NMR spectrum of compound **1**


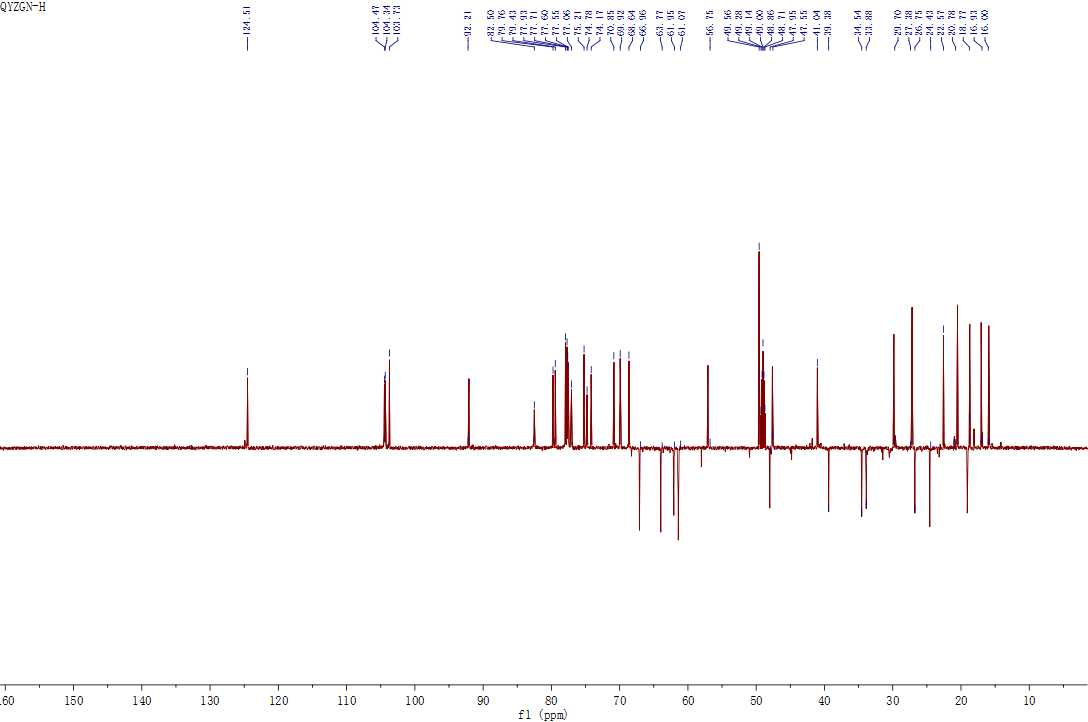
Figure S3. DEPT 135 spectrum of compound 1

#
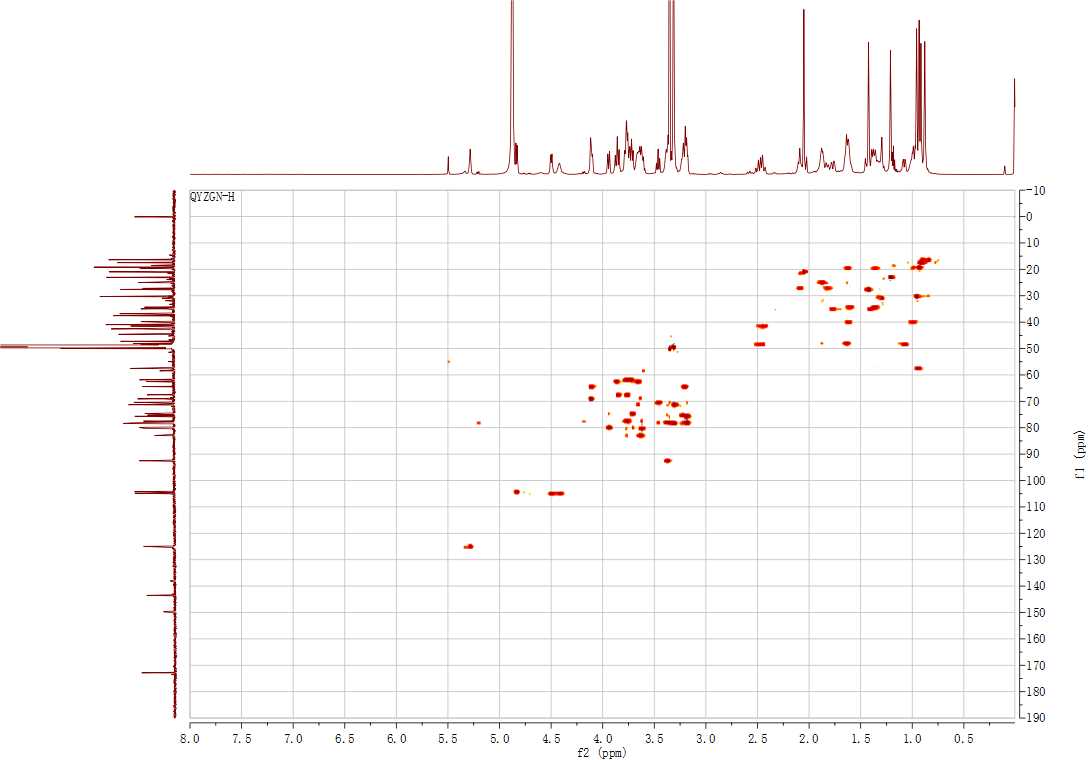


# Figure S4. HSQC spectrum of compound **1**


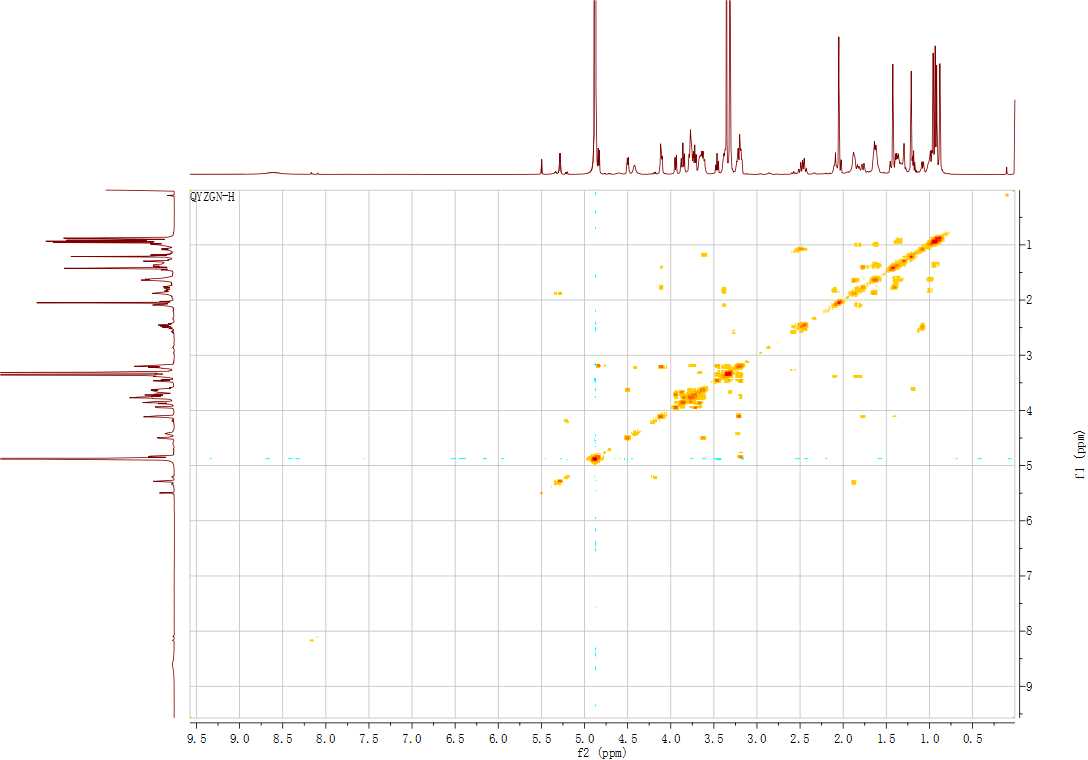


# Figure S5. ^1^H-^1^H COSY spectrum of compound **1**


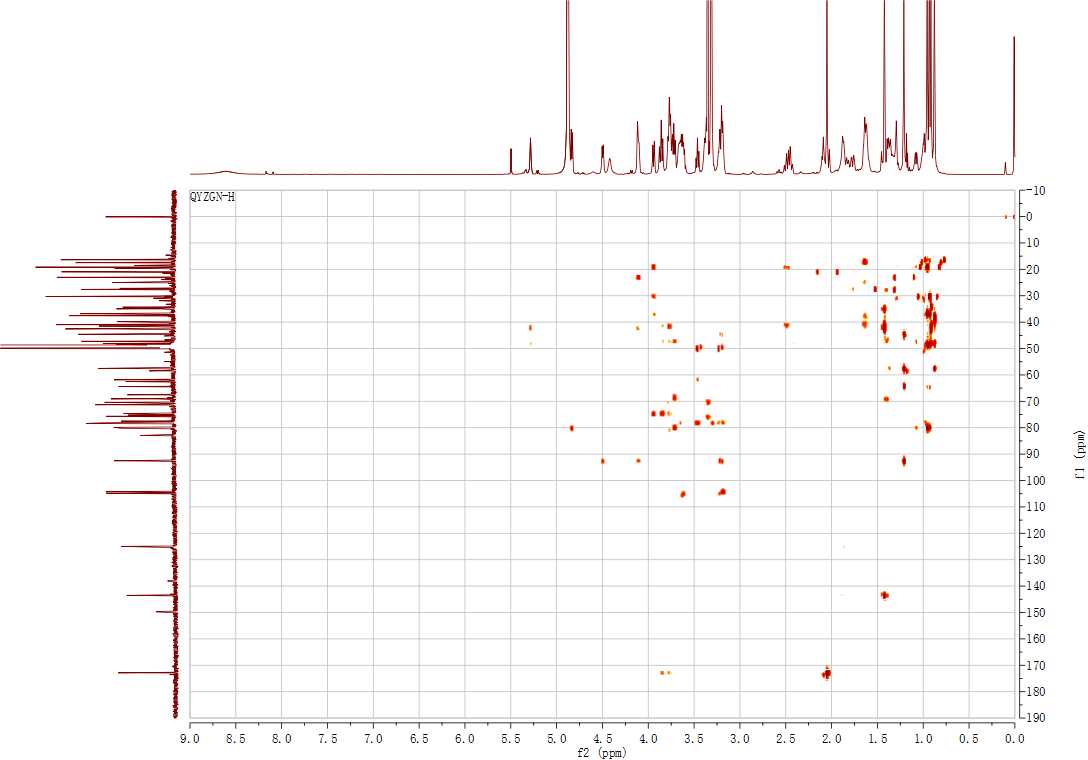


# Figure S6. HMBC spectrum of compound 1


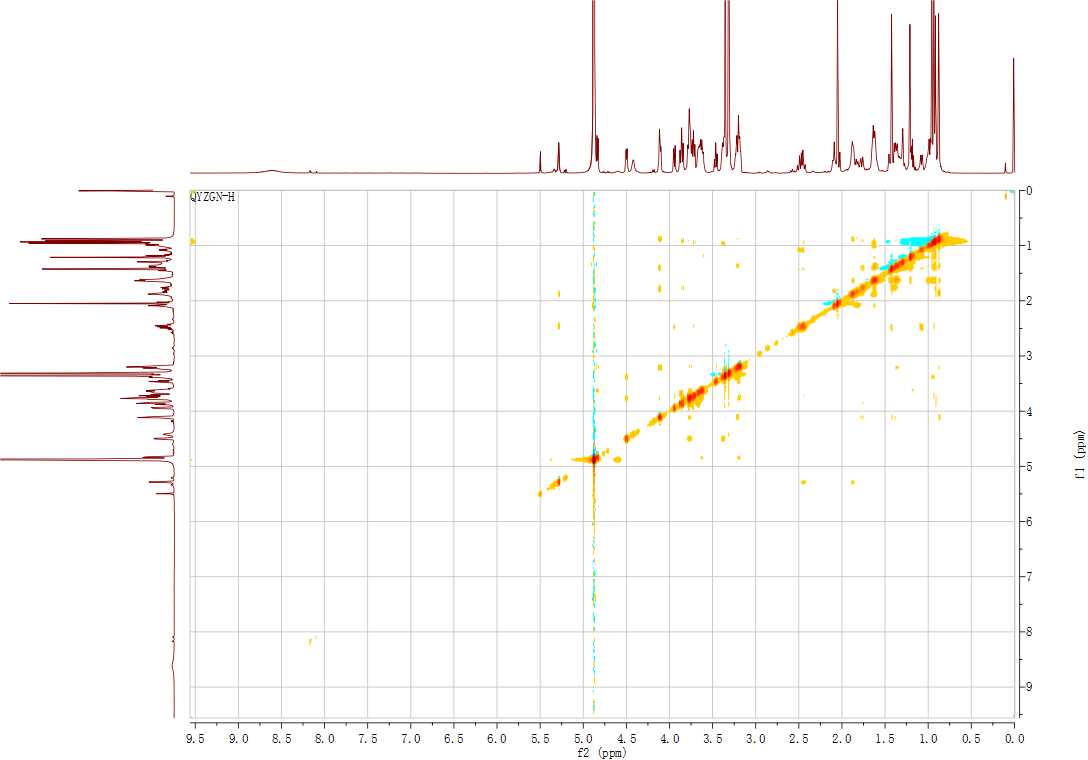


# Figure S7. NOESY spectrum of compound 1

# Figure S8. HR-ESI-MS of compound 1


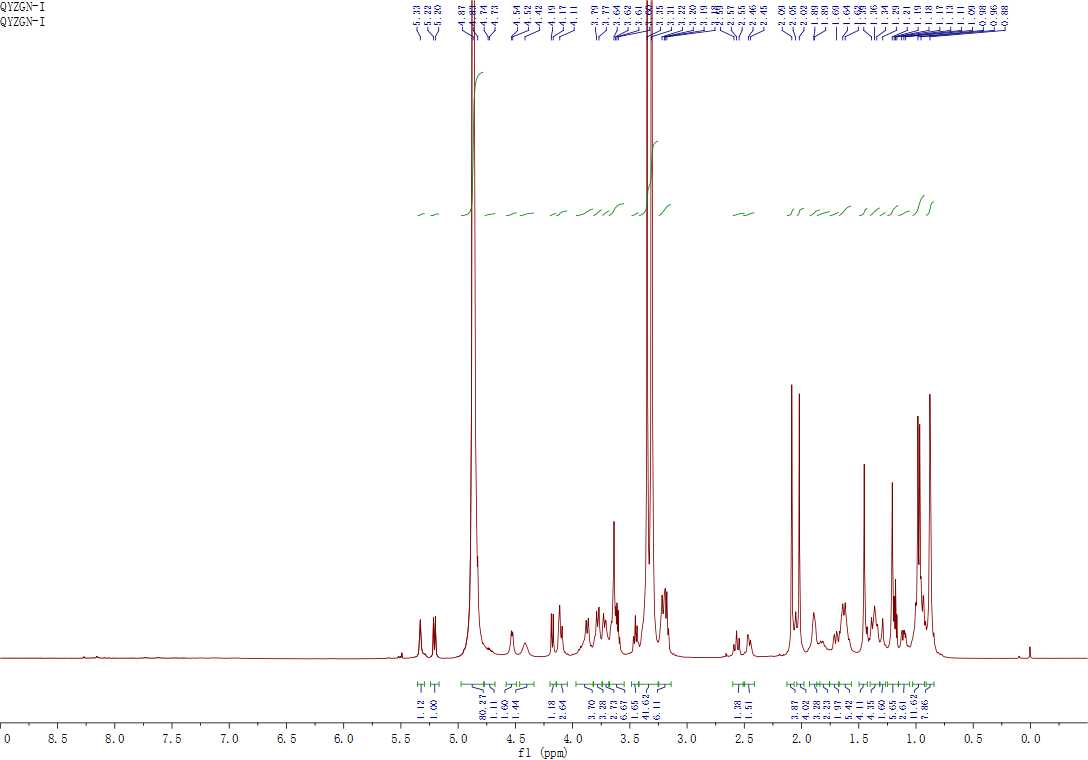


# Figure S9. ^1^H-NMR spectrum of compound 2


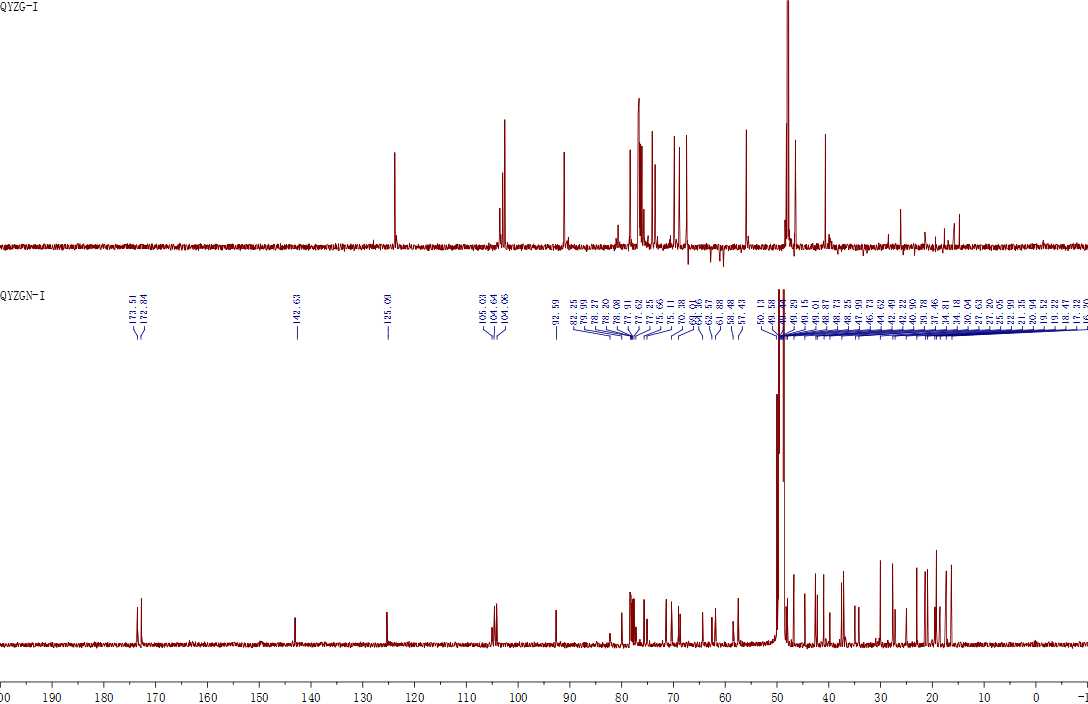


# Figure S10. DEPT 90 and ^13^C-NMR spectrum of compound **2**


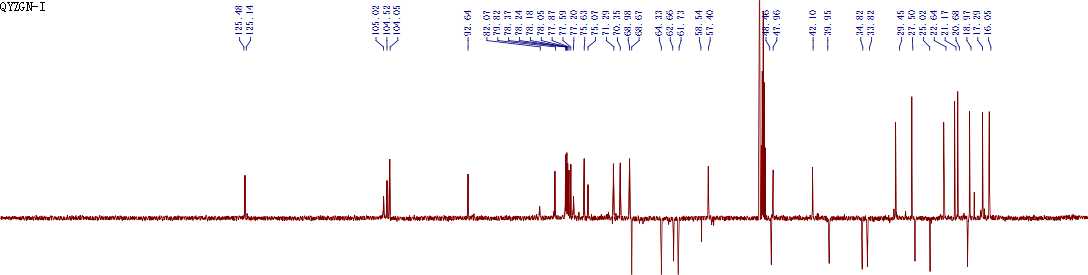


# Figure S11. DEPT 135 spectrum of compound 2

**
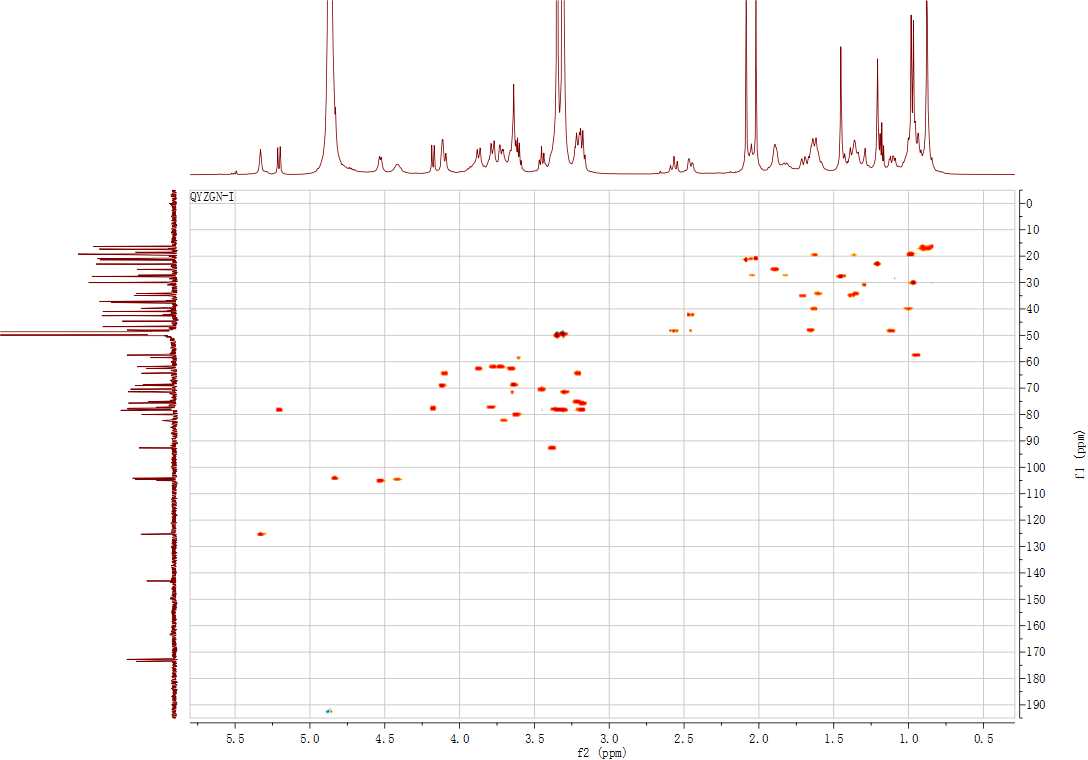
**

# Figure S12. HSQC spectrum of compound 2

**
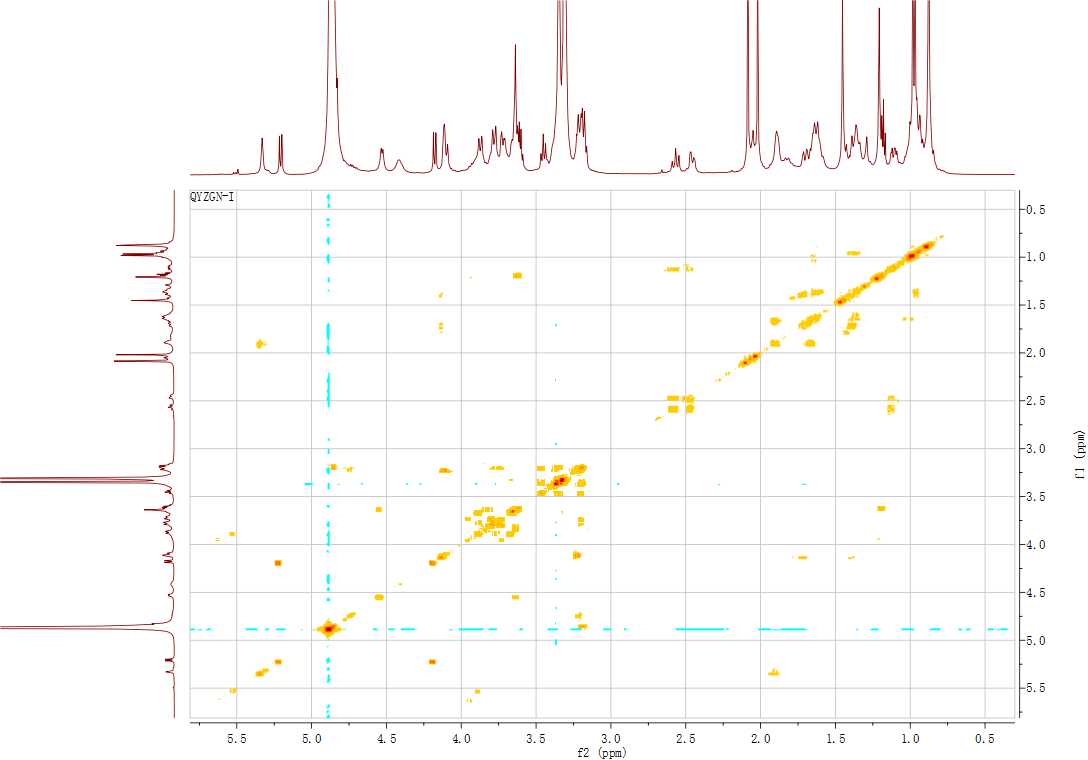
**

# Figure S13. ^1^H-^1^H COSY spectrum of compound **2**

**
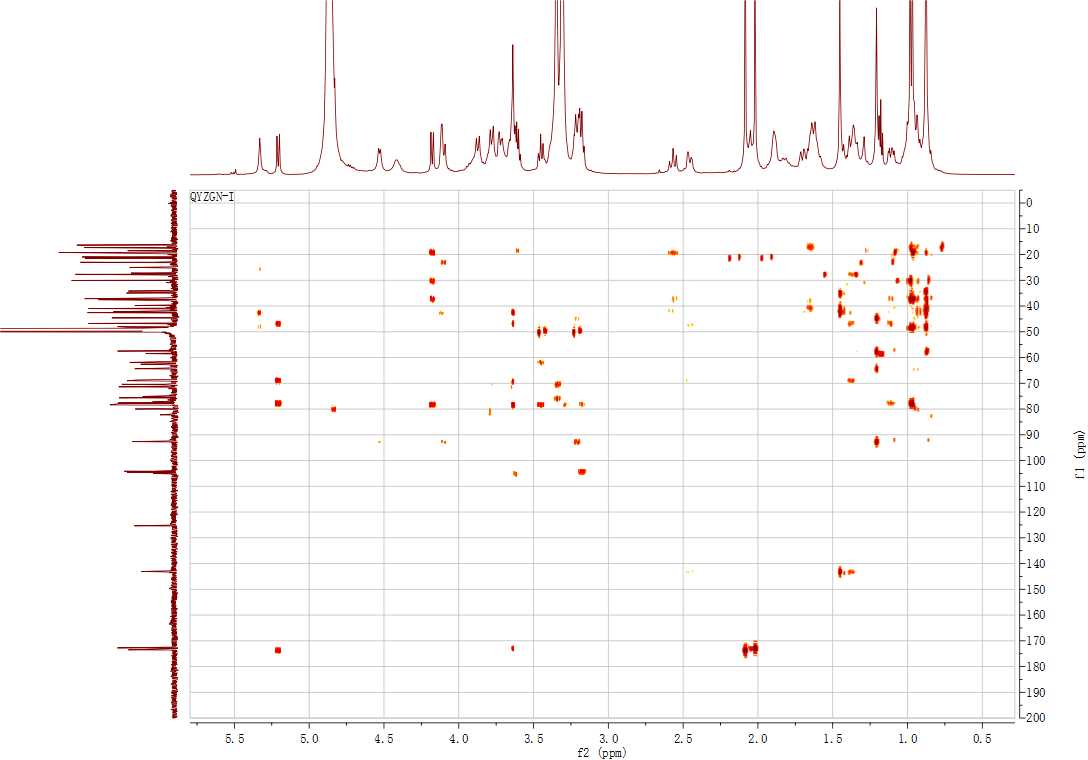
**

# Figure S14. HMBC spectrum of compound 2


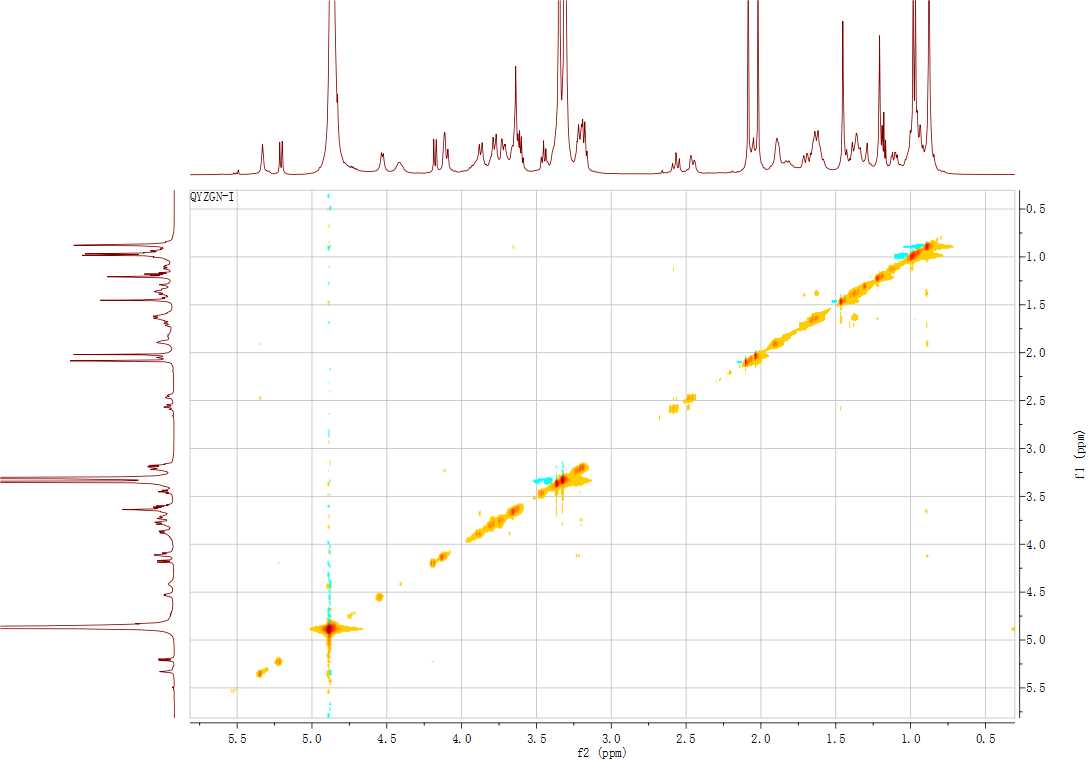


# Figure S15. NOESY spectrum of compound 2

# Figure S16. HR-ESI-MS of compound 2


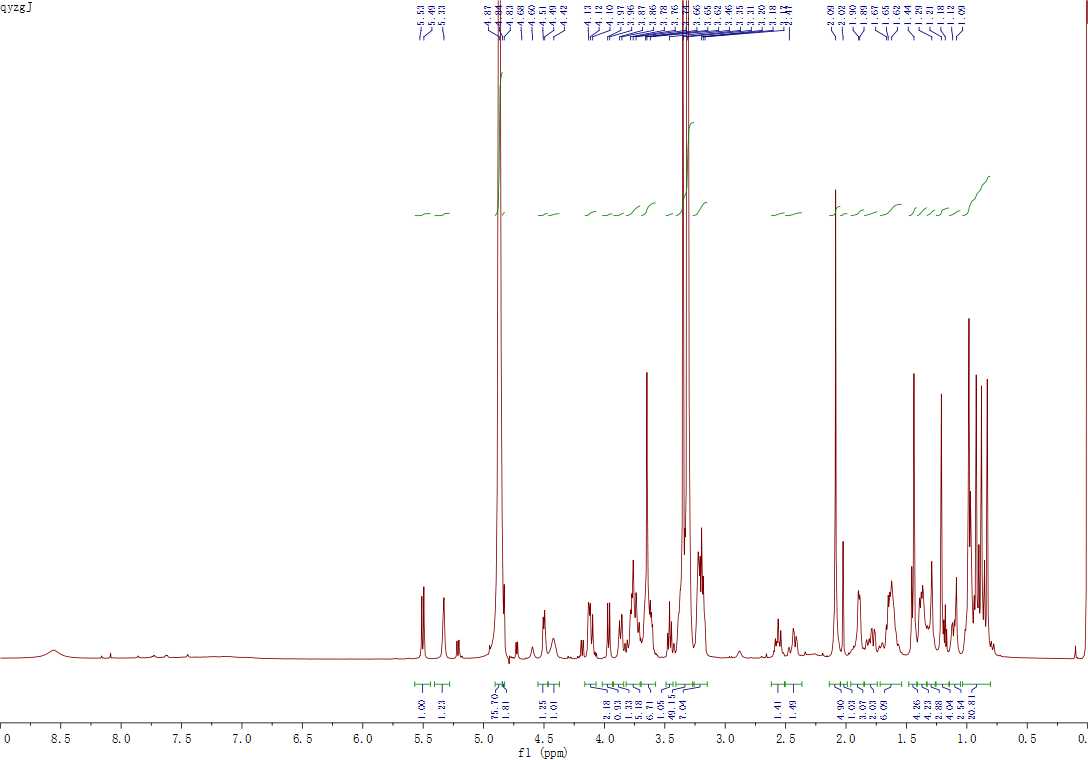


# Figure S17. ^1^H-NMR spectrum of compound **3**

**
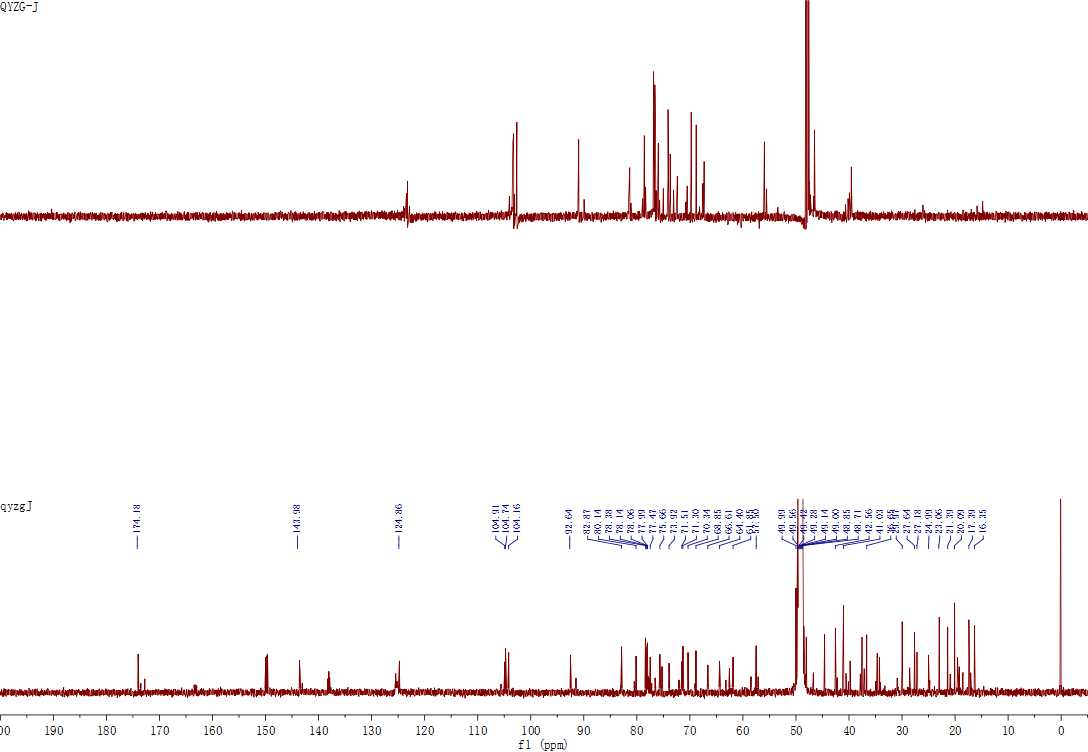
**

# Figure S18. DEPT 90 and ^13^C-NMR spectrum of compound 3

**
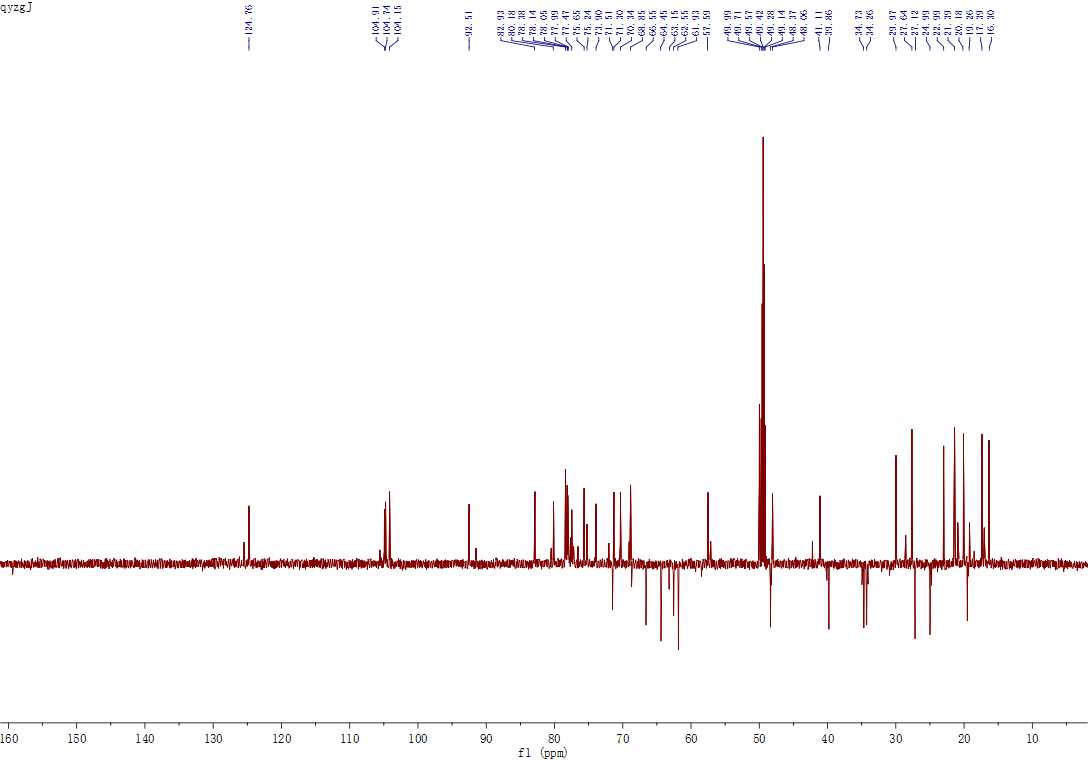
**

# Figure S19. DEPT 135 spectrum of compound 3


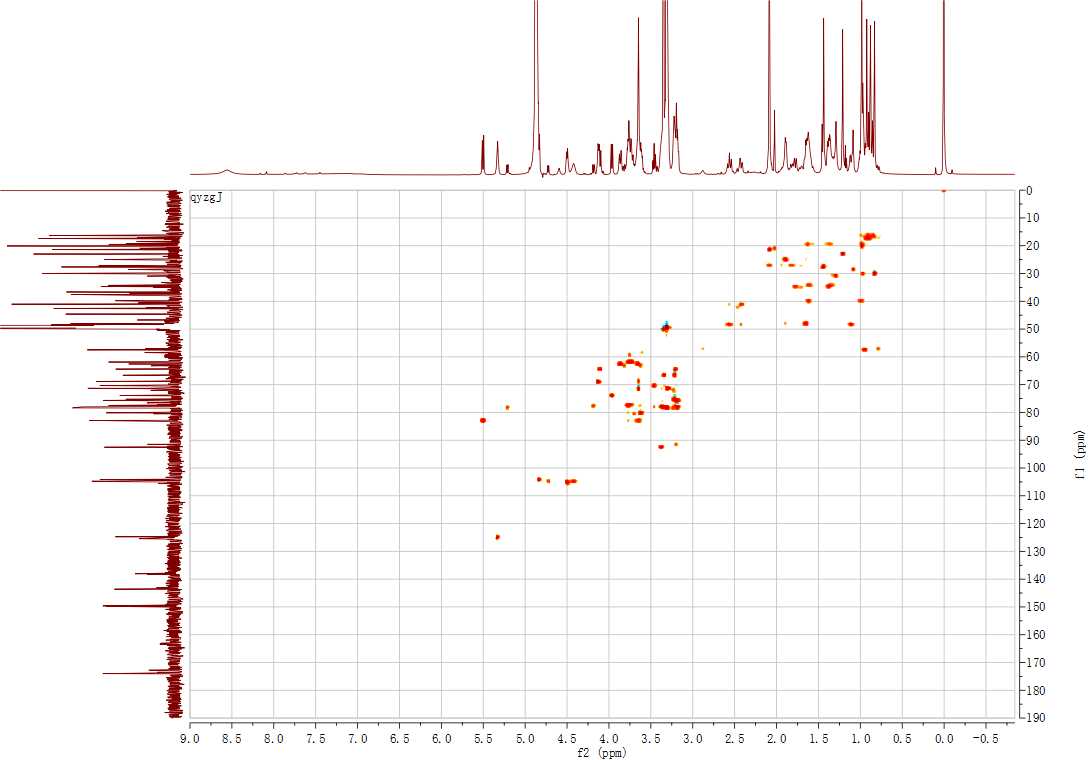


# Figure S20. HSQC spectrum of compound 3

**
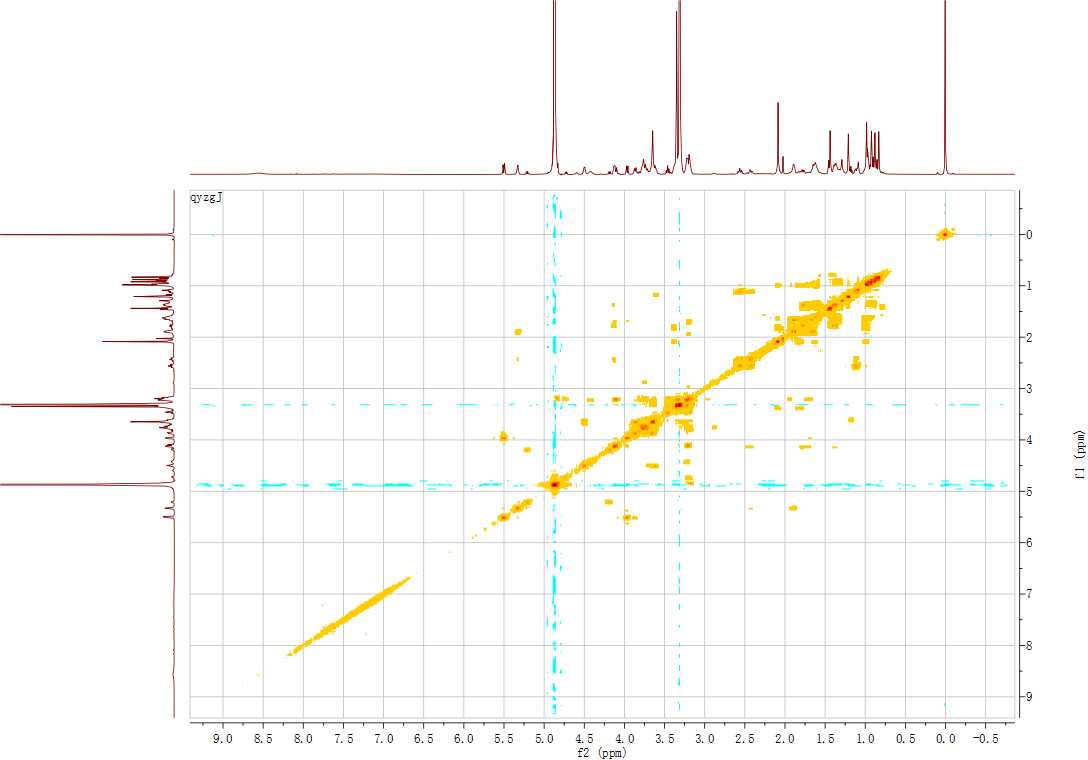
**

Figure S21. ^1^H-^1^H COSY spectrum of compound 3


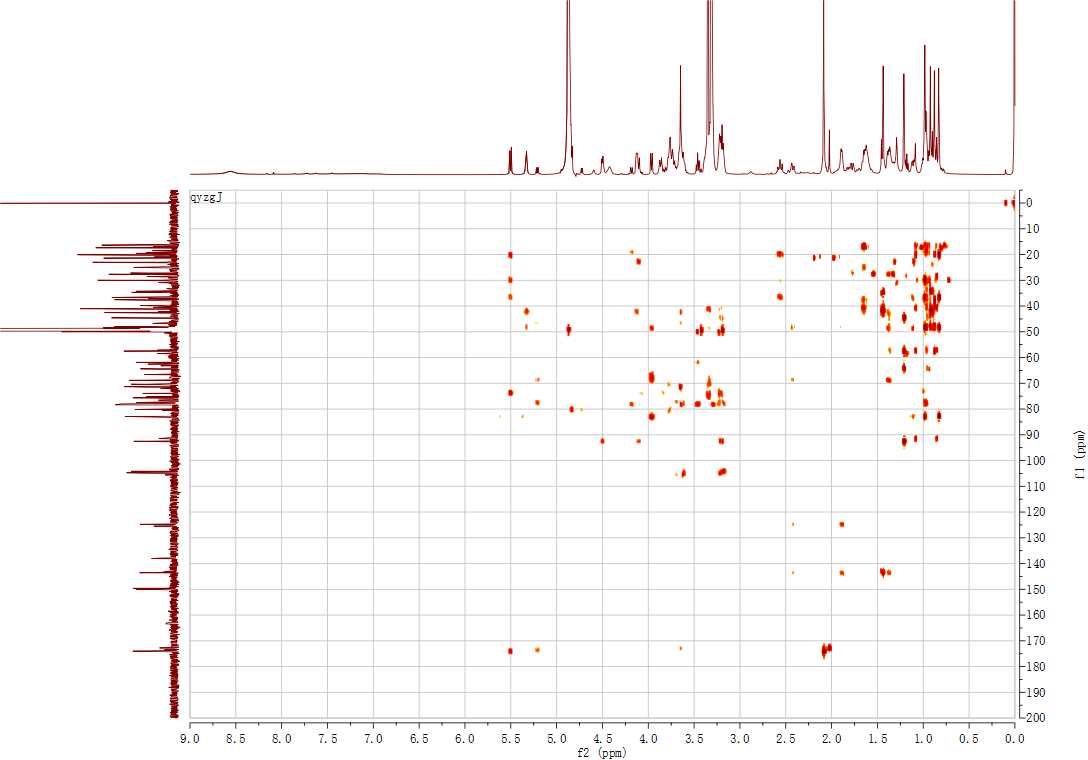


Figure S22. HMBC spectrum of compound 3

**
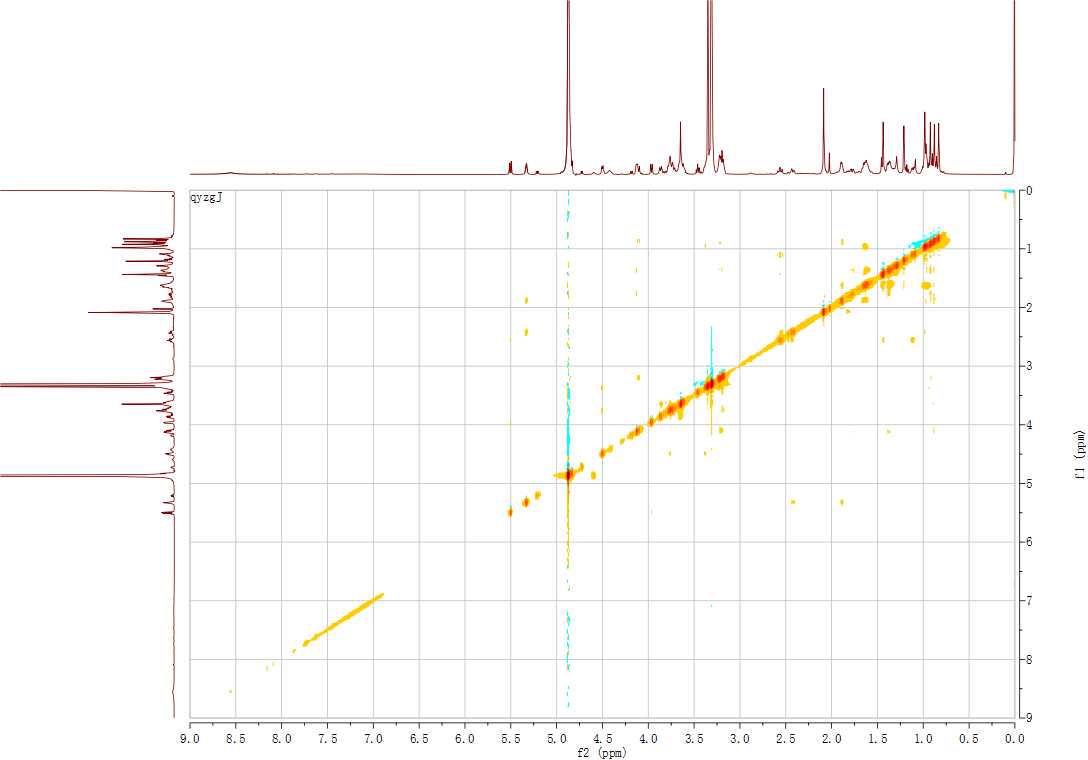
**

# Figure S23. NOESY spectrum of compound 3

# Figure S24. HR-ESI-MS of compound 3

**
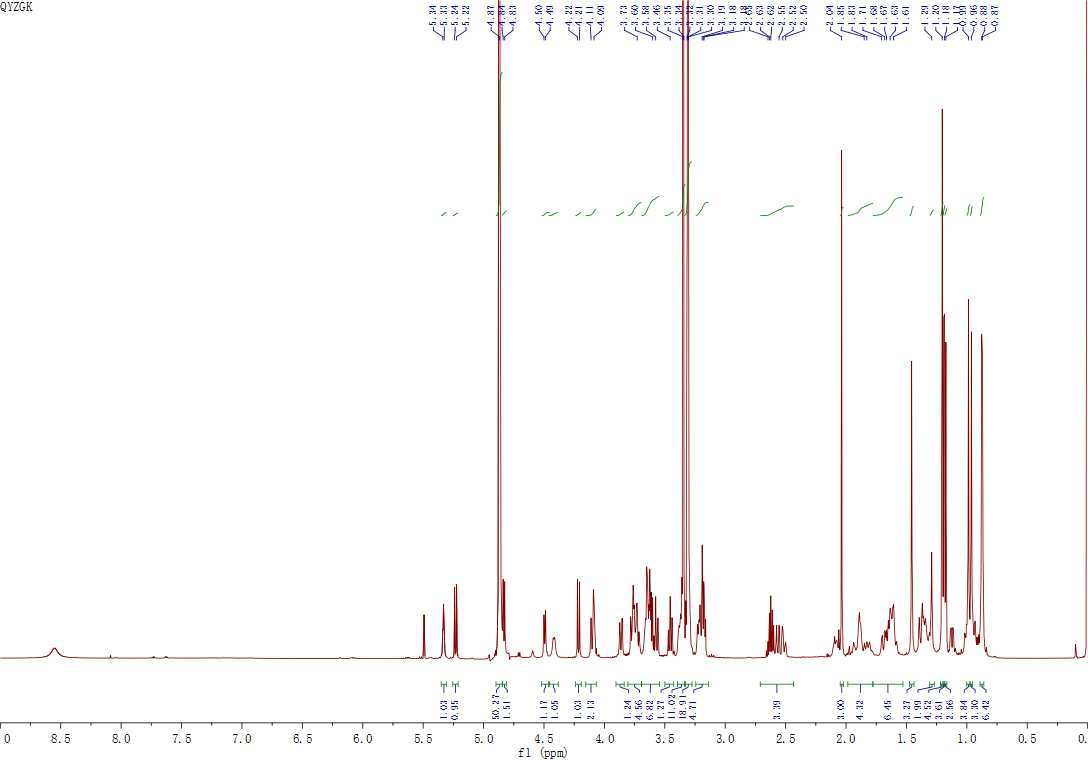
**

# Figure S25. ^1^H-NMR spectrum of compound 4

**
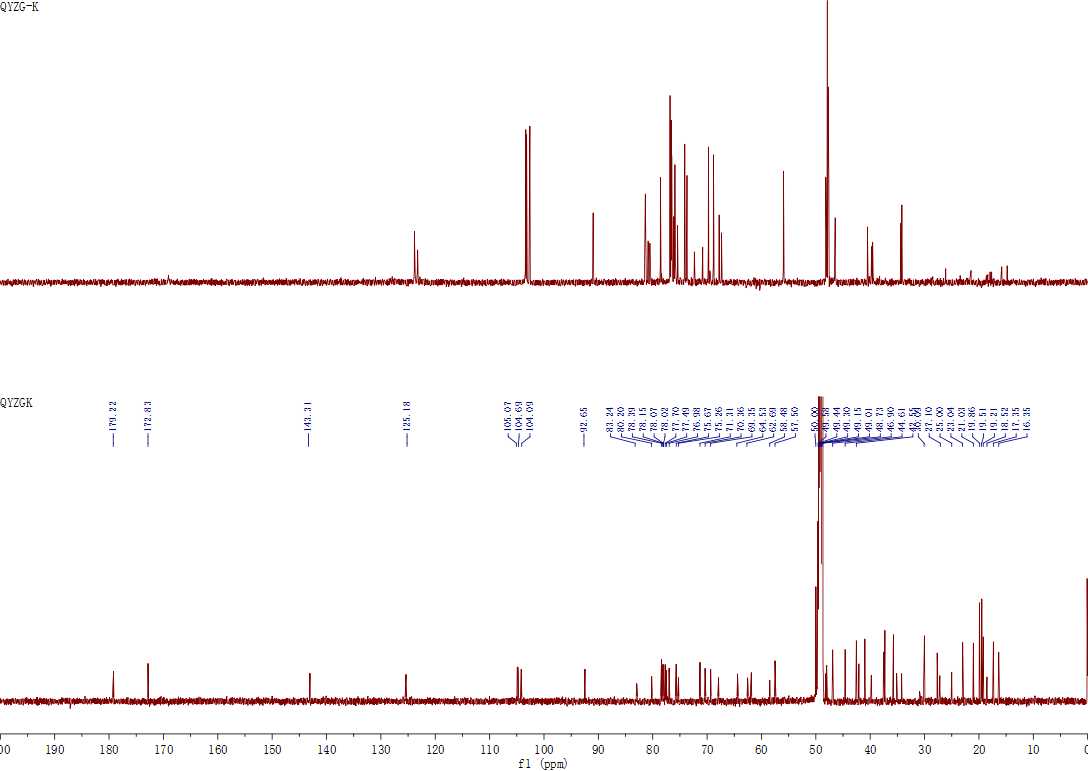
**

# Figure S26. DEPT 90 and ^13^C-NMR spectrum of compound 4

**
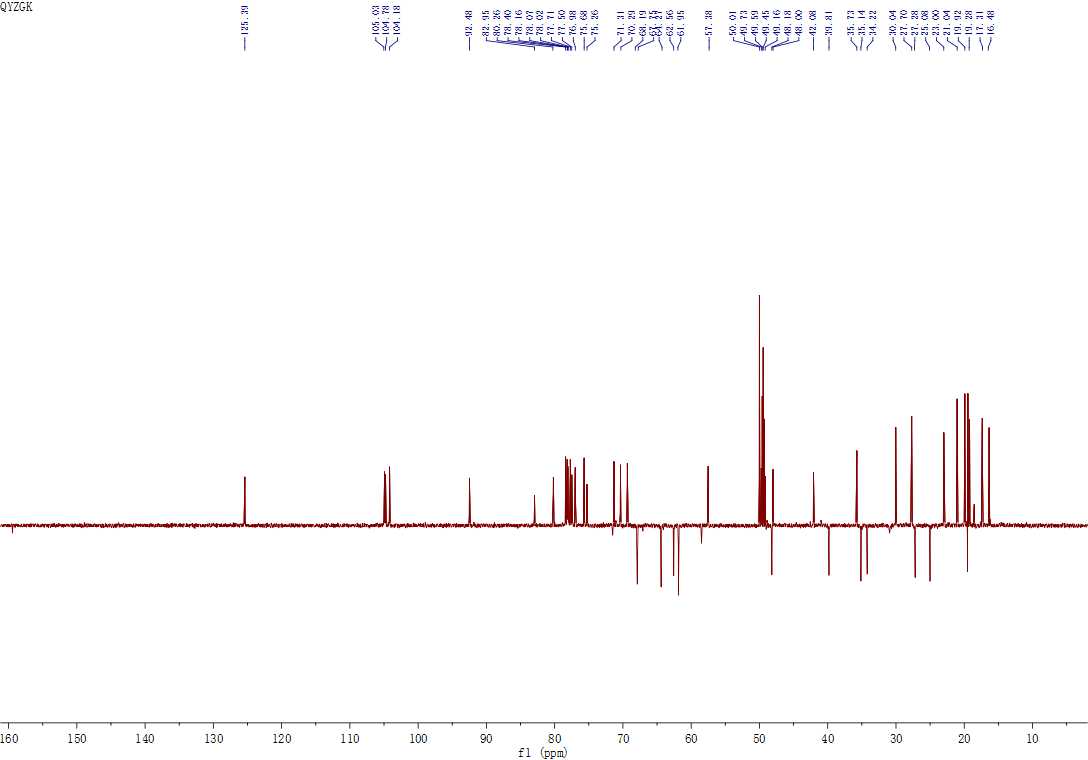
**Figure S27. DEPT 135 spectrum of compound **4**

**
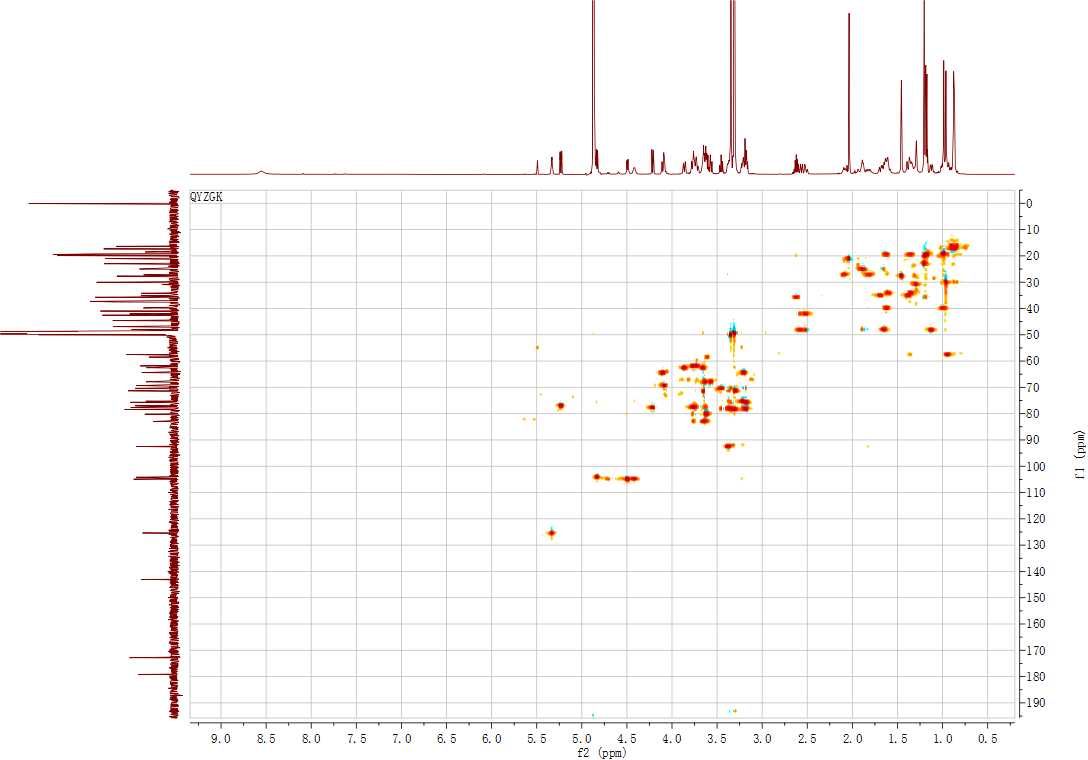
**

# Figure S28. HSQC spectrum of compound 4

**
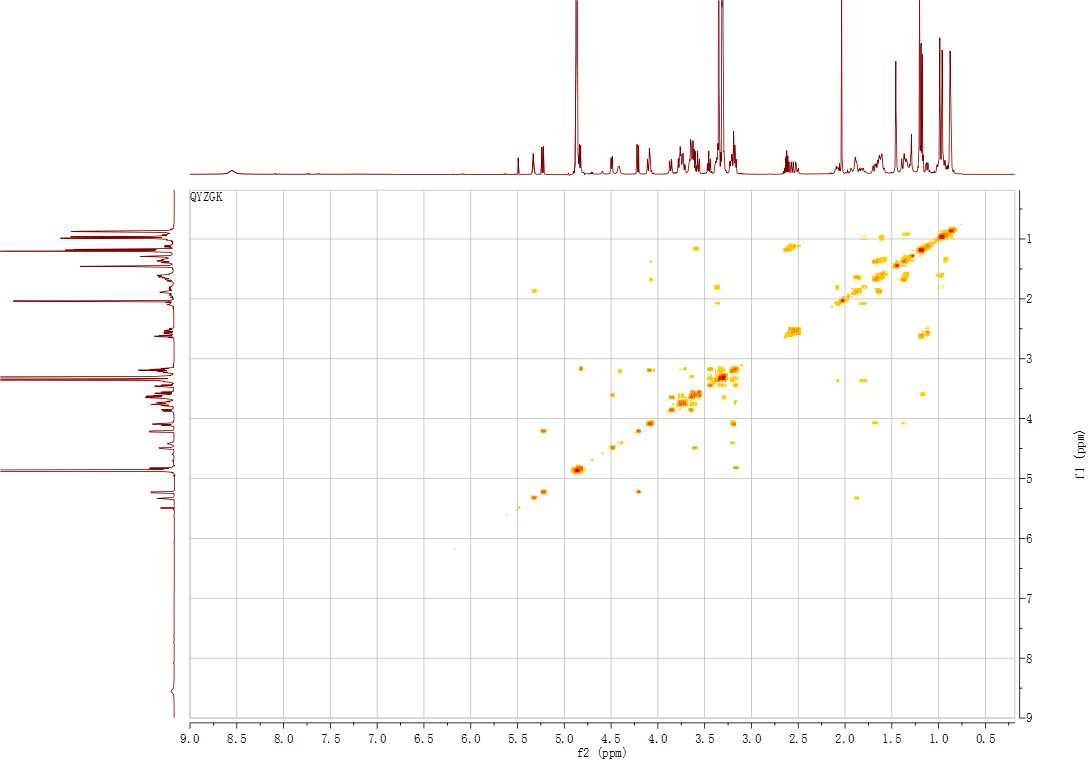
**

# Figure S29. ^1^H-^1^H COSY spectrum of compound 4

**
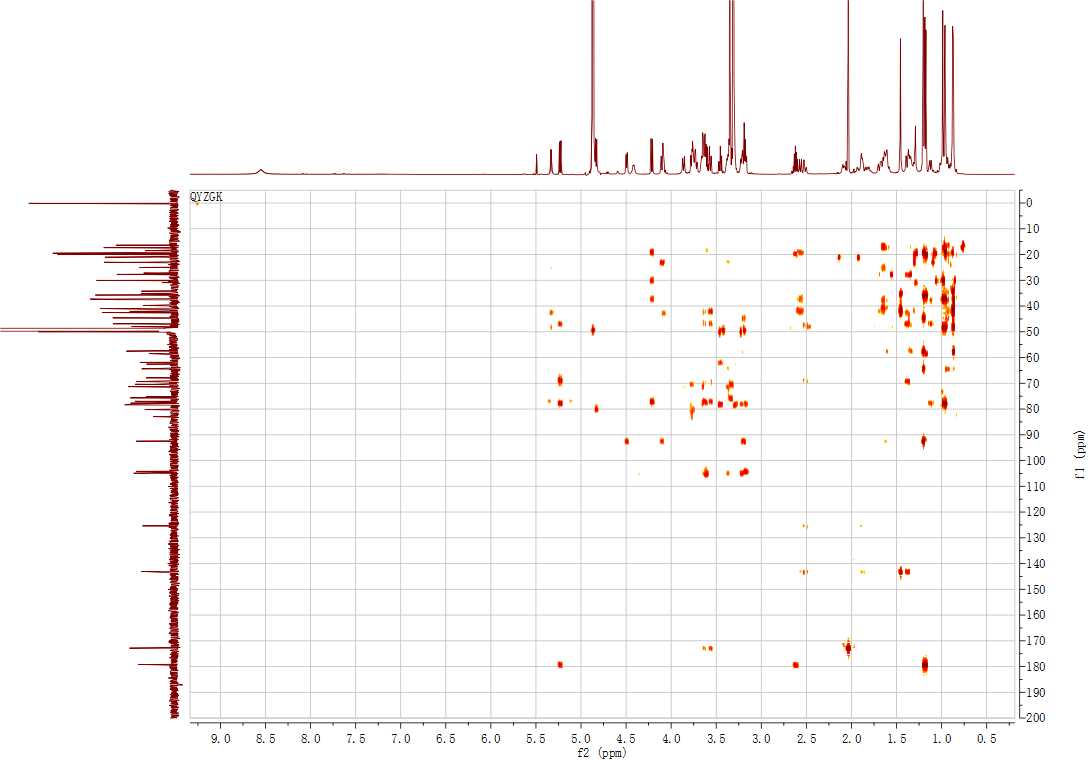
**

# Figure S30. HMBC spectrum of compound 4

# Figure S31. HR-ESI-MS of compound 4


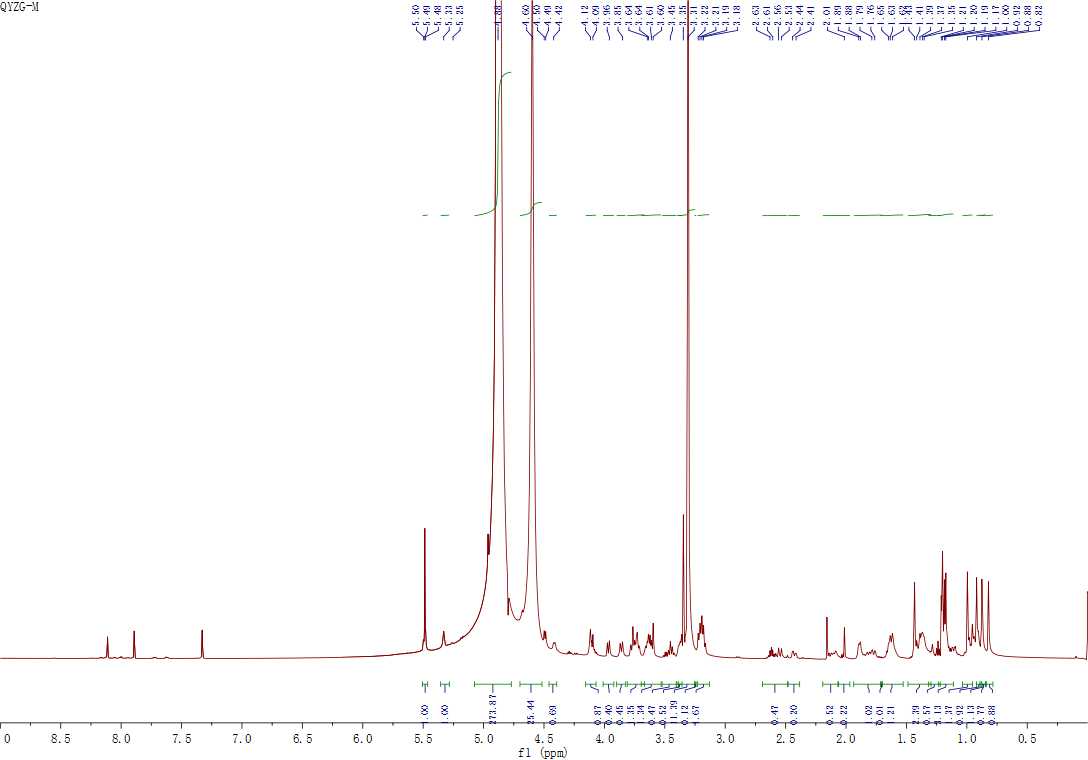


# Figure S32. ^1^H-NMR spectrum of compound 5


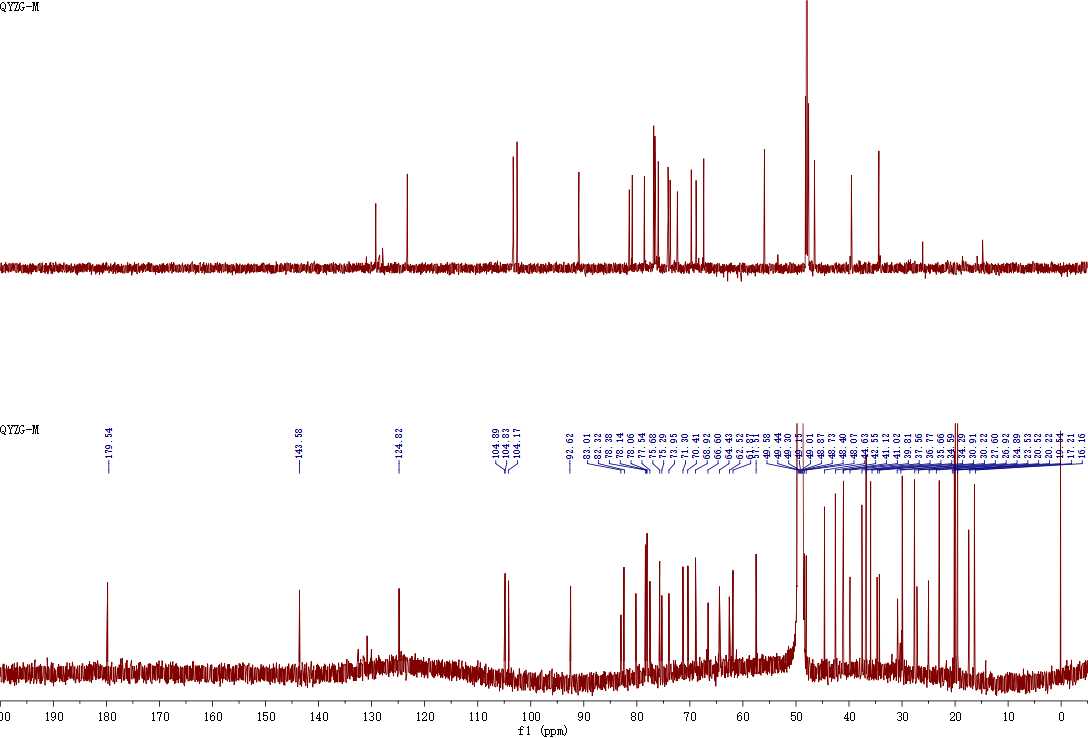


# Figure S33. DEPT 90 and ^13^C-NMR spectrum of compound 5

**
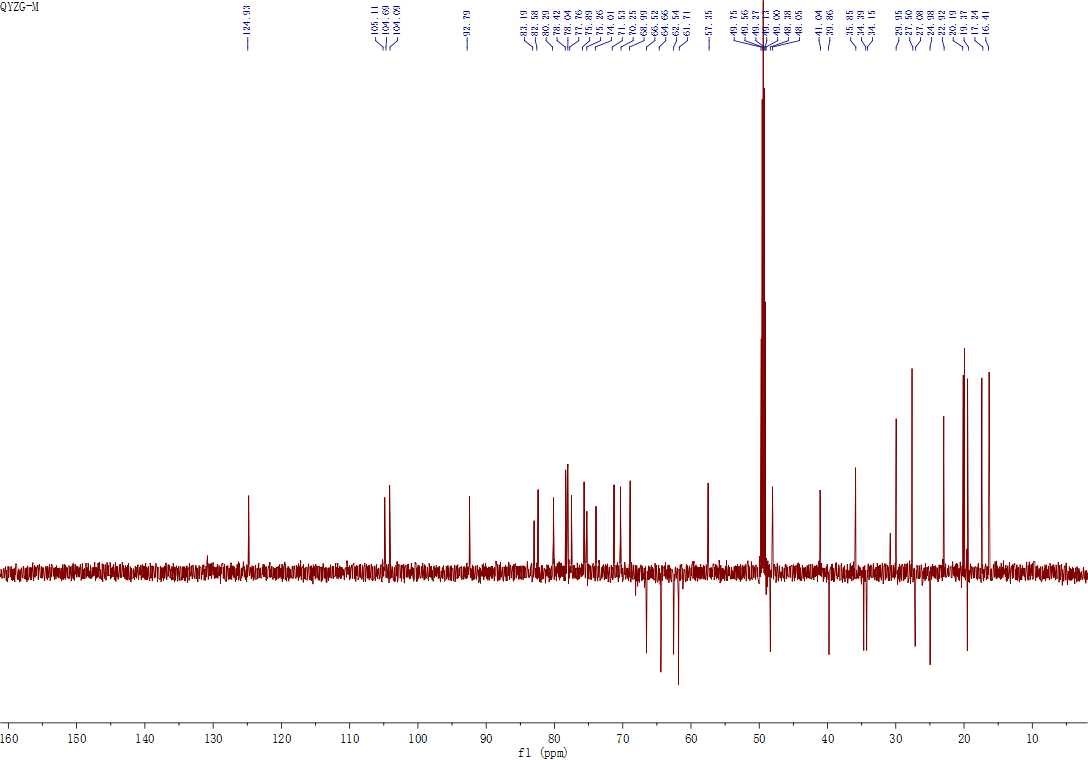
**

Figure S34. DEPT 135 spectrum of compound 5

**
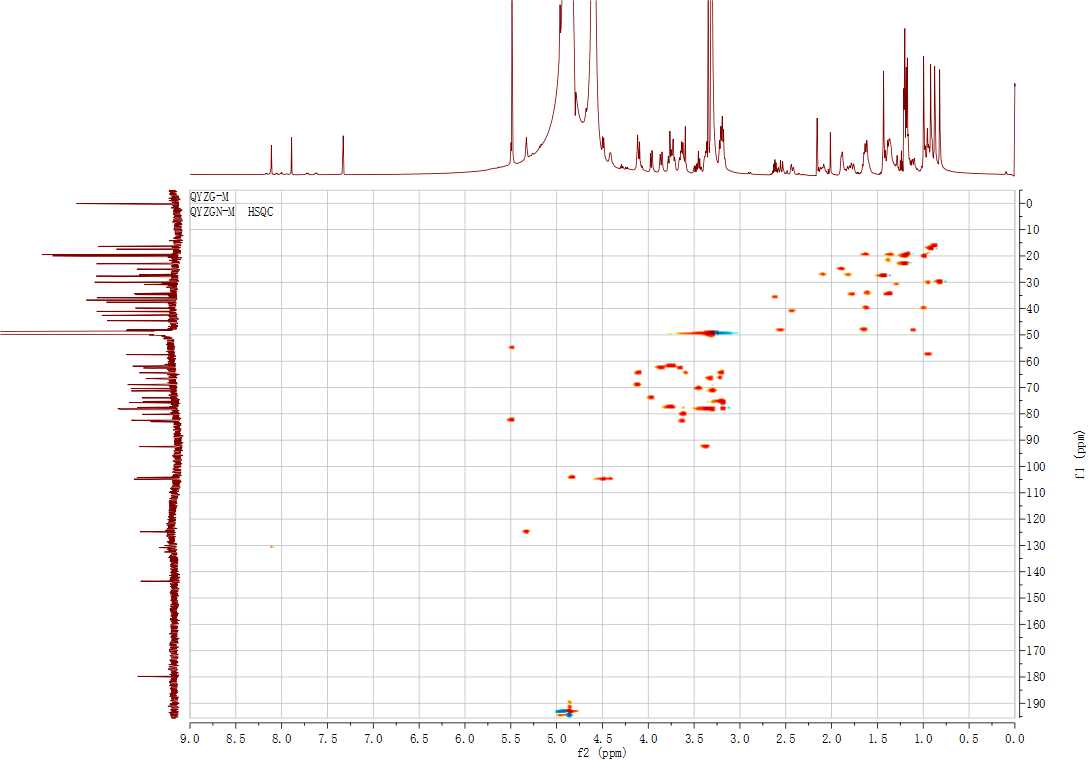
**

# Figure S35. HSQC spectrum of compound 5

**
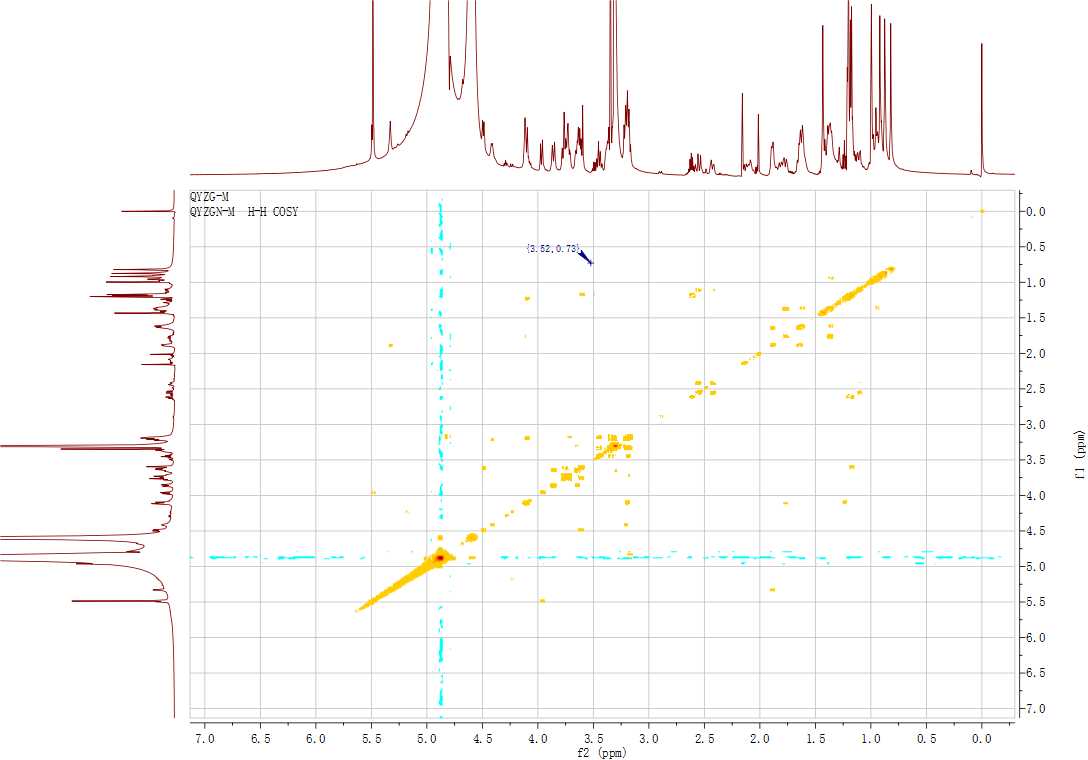
**

# Figure S36. ^1^H-^1^H COSY spectrum of compound 5

**
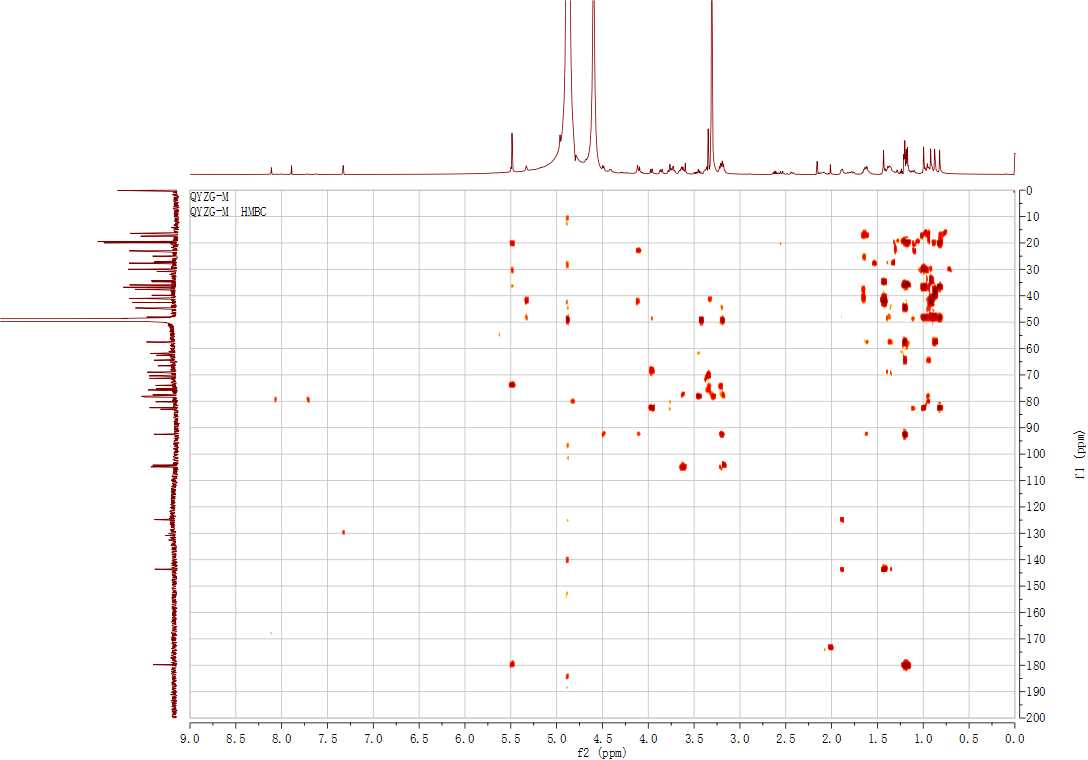
**

# Figure S37. HMBC spectrum of compound 5

**
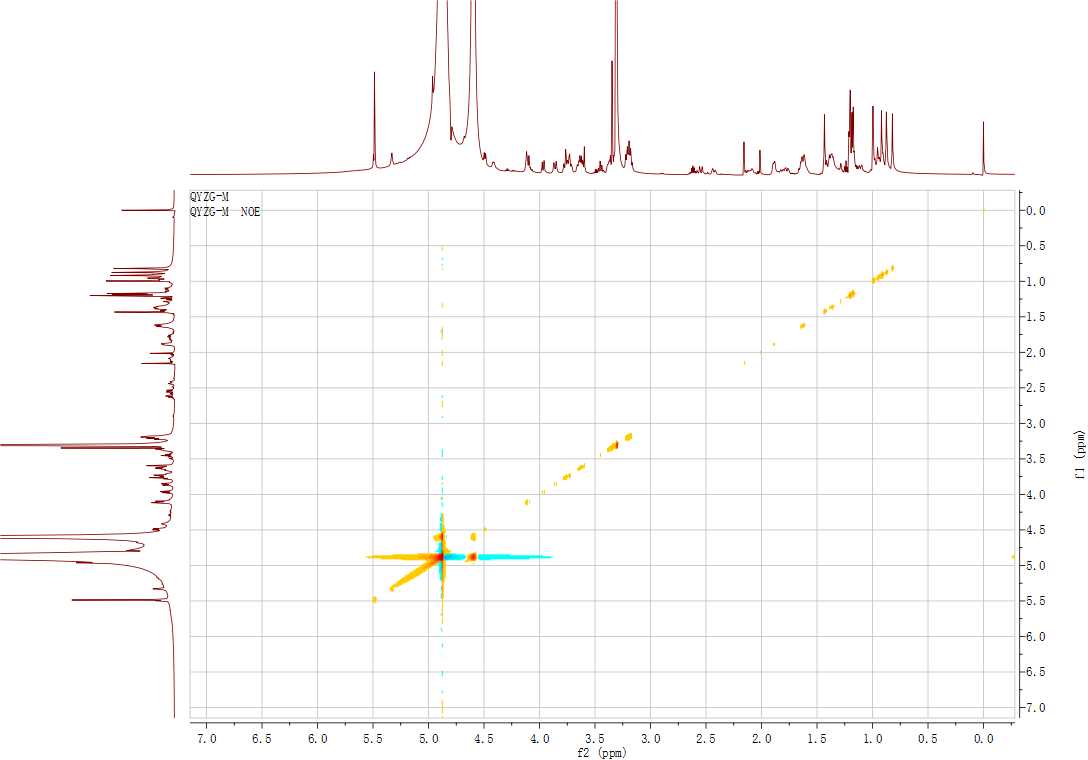
**

# Figure S38. NOESY spectrum of compound 5


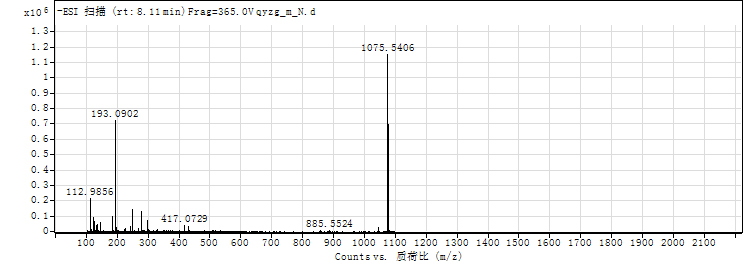


# Figure S39. HR-ESI-MS of compound 5

**
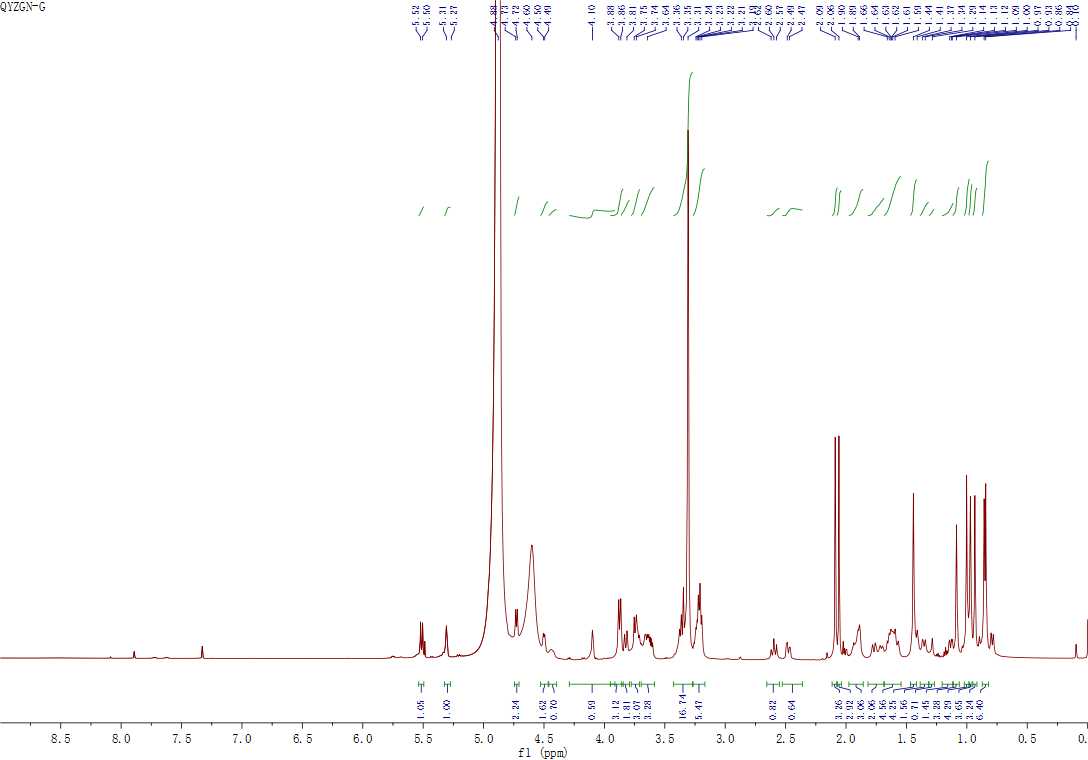
**

# Figure S40. ^1^H-NMR spectrum of compound 6

**
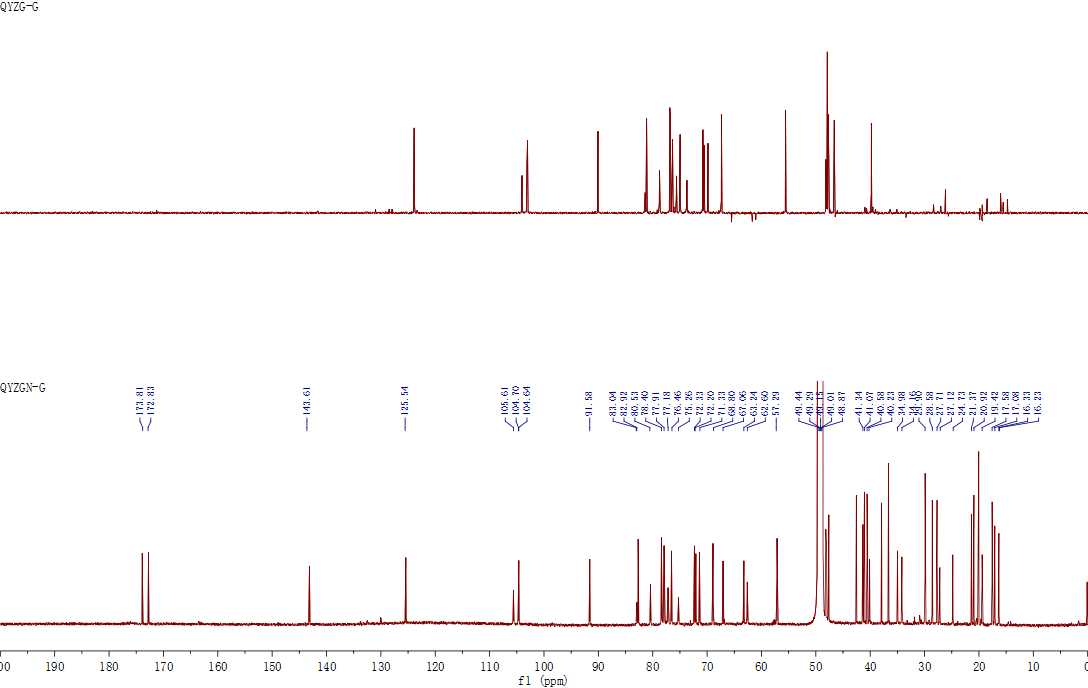
**

# Figure S41. DEPT 90 and ^13^C-NMR spectrum of compound 6


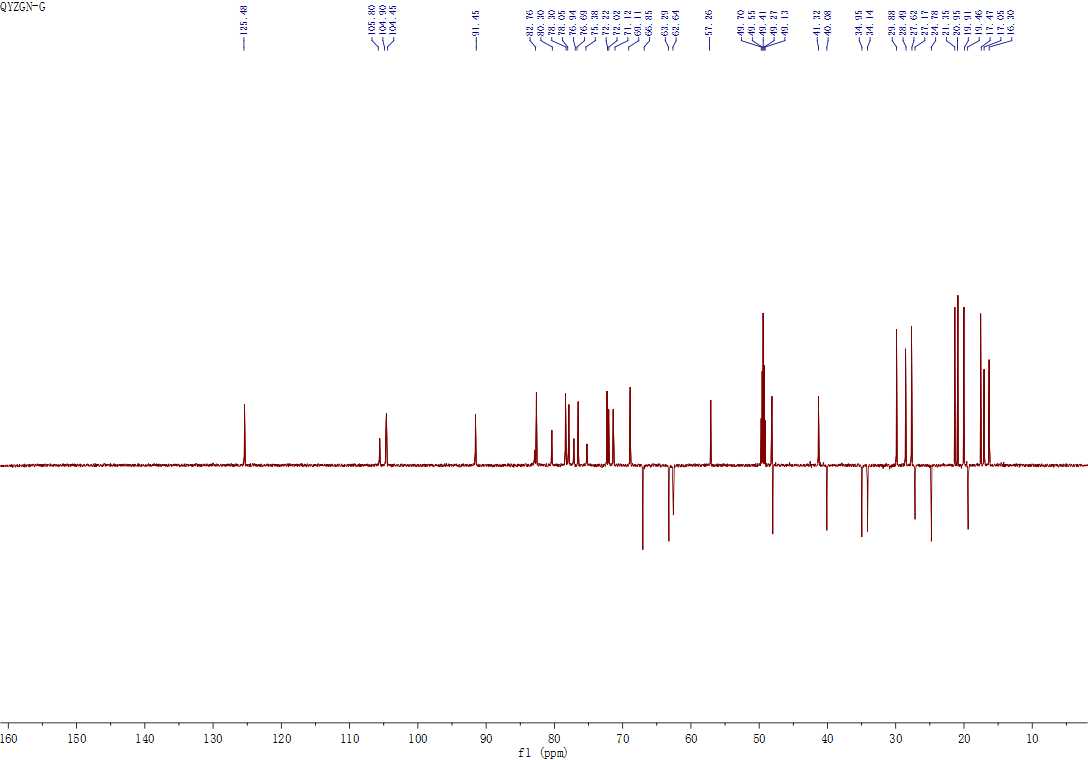


Figure S42. DEPT 135 spectrum of compound 6

**
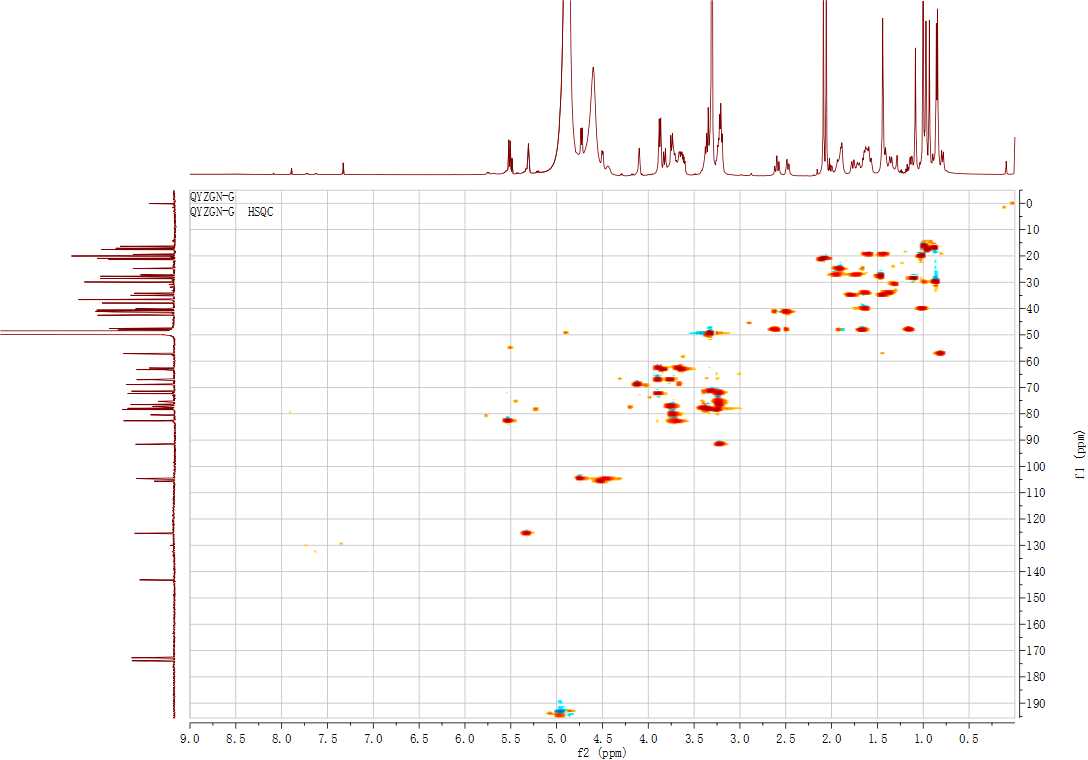
**

# Figure S43. HSQC spectrum of compound 6


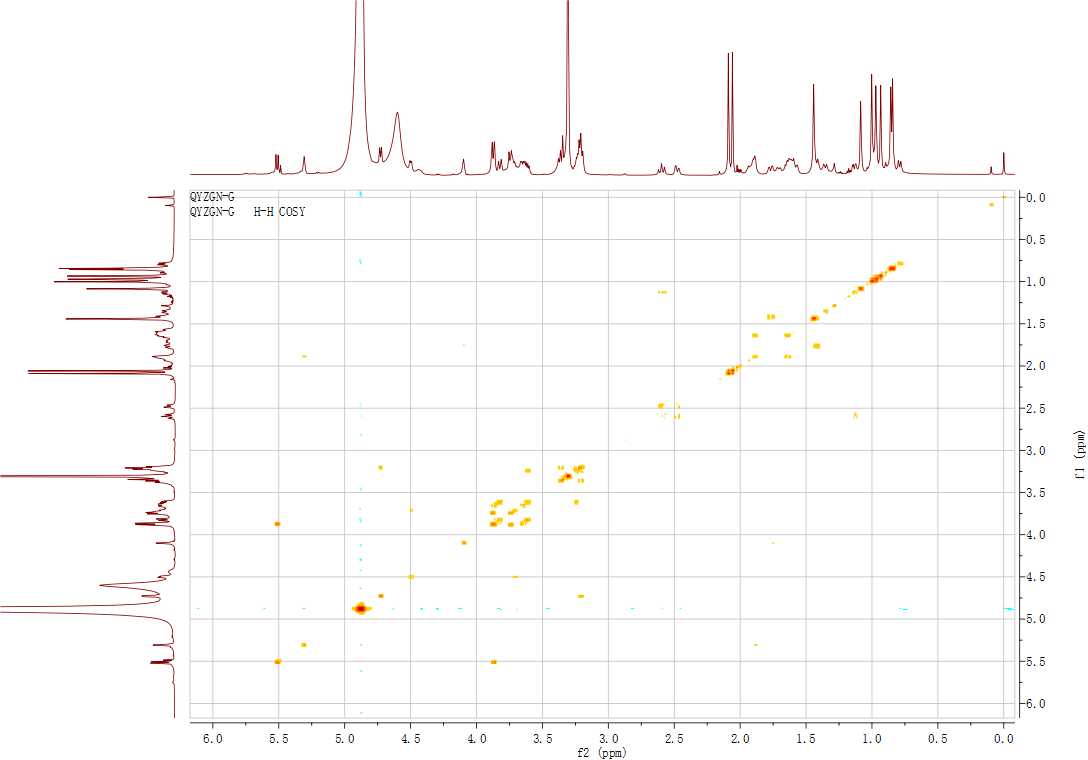


# Figure S44. ^1^H-^1^H COSY spectrum of compound 6


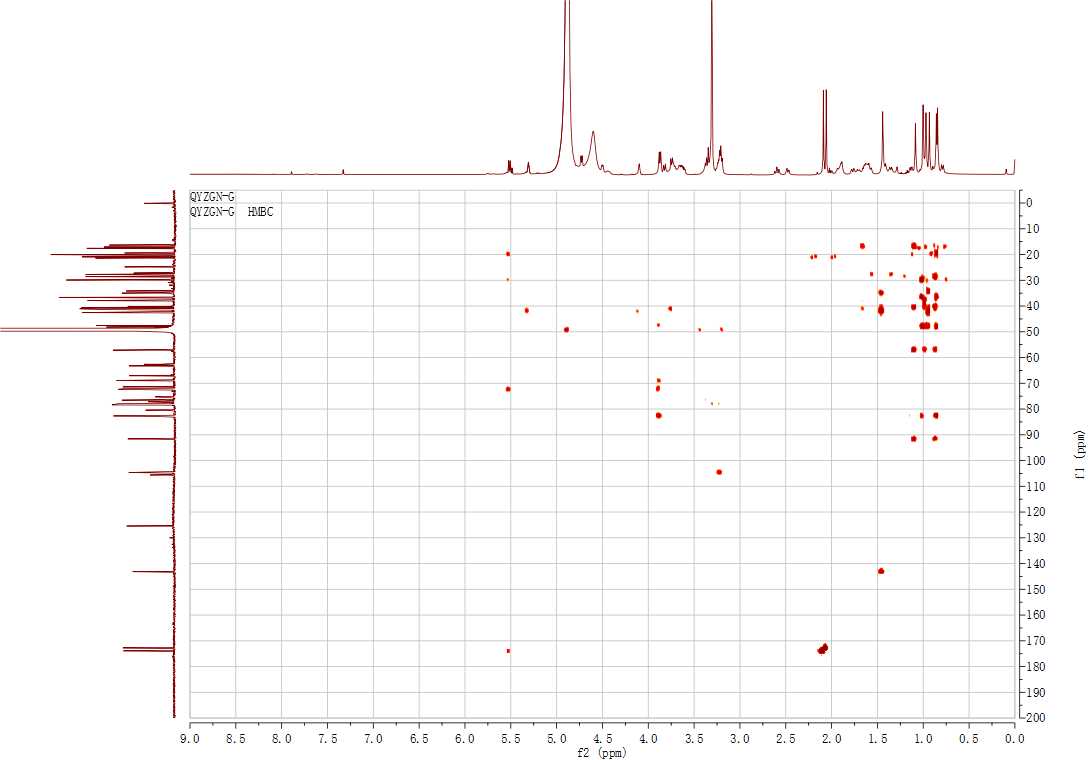


# Figure S45. HMBC spectrum of compound 6


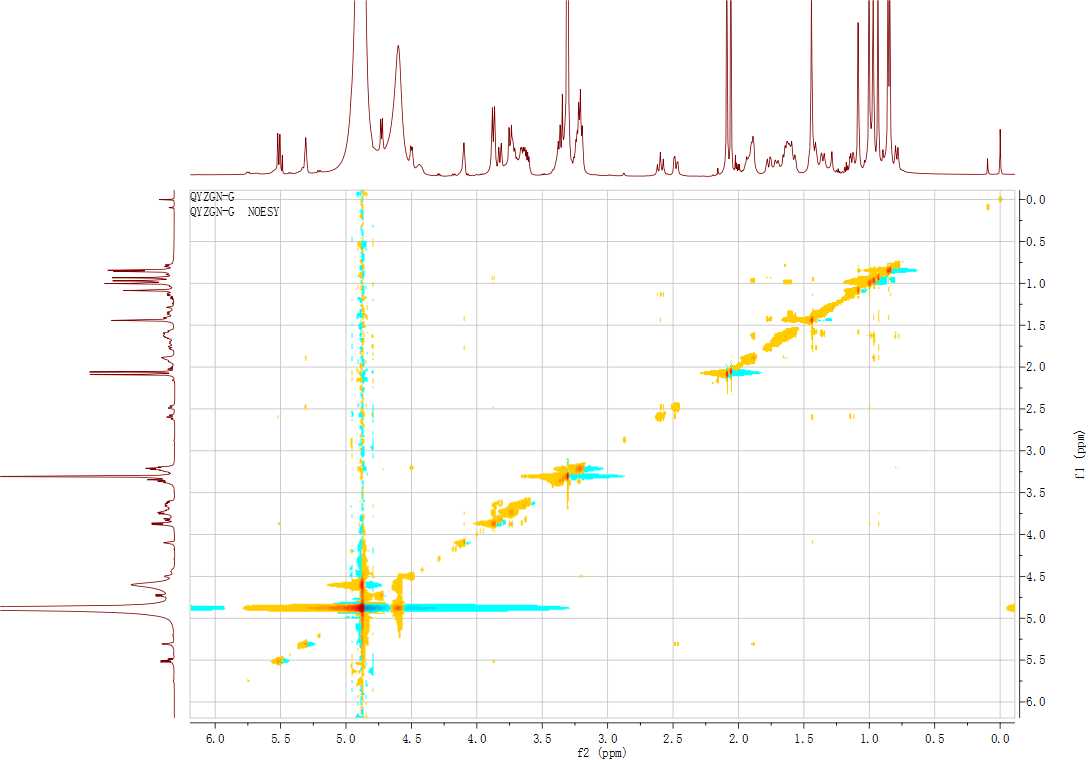


Figure S46. NOESY spectrum of compound 6

# Figure S47. HR-ESI-MS of compound 6
